# The population structure of *Acinetobacter baumannii* isolated from animals: an emerging zoonotic threat

**DOI:** 10.1128/aem.00038-26

**Published:** 2026-06-15

**Authors:** Xiangkuan Zheng, Peifan Liu, Xin Wang, Huanxin Fang, Haiyan Zhang, Shuang Zhang, Yishan Zheng, Wei Zhang

**Affiliations:** 1 College of Veterinary Medicine, Nanjing Agricultural University 70578, Nanjing, People's Republic of China; 2 Key Lab of Animal Bacteriology, Ministry of Agriculture and Rural Affairs, Nanjing, People's Republic of China; 3 WOAH Reference Lab for Swine Streptococcosis, Nanjing, People's Republic of China; 4 Department of Food and Biology Engineering, Wuhu Institute of Technology 530470, Wuhu, People's Republic of China; 5 Intensive Care Unit, The Second Hospital of Nanjing, Nanjing University of Chinese Medicine 66478, Nanjing, People's Republic of China; Universite de la Reunion, Ste Clotilde, France

**Keywords:** *Acinetobacter baumannii*, population structure, host, antimicrobial resistance, zoonosis

## Abstract

To investigate the population structure of *Acinetobacter baumannii* in animals and the transmission pattern between humans and animals, we conducted a molecular epidemiological study by supplementing the data from poultry combined with data from 509 animal isolates and 2,218 human isolates in the NCBI GenBank database. Antibiotic susceptibility testing and genomic analysis showed that *A. baumannii* isolated from the brains of dead poultry embryos was universally sensitive to 19 antibiotics and genetically diverse. The pathogen has been isolated from 33 animal species from 17 countries worldwide. According to the established international clones (ICs), IC2 (*n* = 2), IC5 (*n* = 9), IC7 (*n* = 6), and IC8 (*n* = 10) were the main clonal lineages among the 170 sequence types (STs). Particularly, the ST25 lineage isolates in IC7, which were detected in nine animal species, mainly companion animals such as cats, dogs, and horses. In addition, 115 STs belonged to unique clones. *A. baumannii* isolated from animals can be divided into two groups: a high antimicrobial resistance (AMR) group dominated by hosts such as horses, cats, and dogs and a low AMR group dominated by hosts such as livestock, poultry, and wildlife. In the low AMR group, the isolates carried fewer AMR and virulence genes than human isolates in the same region. Compared with the low AMR group, isolates in the high AMR group shared more STs with human isolates in the same region. In conclusion, our study suggests that *A. baumannii* has a higher potential of intertransmission between companion animals and humans compared to livestock, poultry, and wildlife.

**IMPORTANCE:**

The population structure of *A. baumannii* in animals has been systematically and comprehensively revealed by molecular epidemiological studies of *A. baumannii* isolated from animals worldwide. *A. baumannii* isolated from animals can be divided into two groups, a high antimicrobial resistance (AMR) group dominated by hosts such as horses, cats, and dogs and a low AMR group dominated by hosts such as livestock, poultry, and wildlife. The integration of the distribution of AMR and virulence genes, the phylogenetic tree, and the multi-locus sequence typing results suggests a higher potential of intertransmission between companion animals and humans. This elevated potential may be related to the close contact of companion animals with humans.

## INTRODUCTION

*A. baumannii* is notorious primarily for causing nosocomial infections and multi-antimicrobial resistance (AMR) (1), leading to conditions such as meningitis (2), bacteremia (3), and pneumonia (4) in humans. The acquired AMR, in conjunction with the spread of its predominant clones, poses a considerable challenge to the effective treatment of infections, thereby underscoring its significance as a major global concern in the fields of healthcare and public health (5, 6).

However, in recent years, the pathogen has been isolated from a wide range of animals and has caused severe infections and deaths in animals (7). The pathogen causes symptoms in infected animals that are similar to those in infected humans, such as causing pneumonia, urinary tract infections, and septicemia in cats and dogs (8–11), respiratory infections and septicemia in horses (12), and meningitis and pneumonia in sheep (13, 14). Furthermore, *A. baumannii* has been isolated from other animals, including swine, cattle, mink, and falcons (15). Some *A. baumannii* isolates from animals exhibit multiple AMR, such as some isolates isolated from cats and dogs, but more seem to exhibit low AMR. *A. baumannii* is also widely present in other non-animal sources, such as food, various water bodies, plants, and soil (16–20). To combat multi-antimicrobial resistant human infection caused by *A. baumannii*, a One Health approach is required, and a deeper understanding of non-human-clinical isolates must be achieved.

In order to study the epidemiology of *A. baumannii*, several molecular typing methods have been developed and have played a key role in the study of the global epidemic of *A. baumannii*. In accordance with the results of repPCR, isolates that are widely prevalent around the world were clustered into eight clonal lineages, which were ultimately named using the term international clones (ICs) 1–8 (21). With the iteration of molecular typing techniques, the “Oxford” and “Pasteur” schemes, two seven-locus multi-locus sequence typing (MLST) schemes for *A. baumannii*, have replaced repPCR as a valuable tool for analyzing the structure of *A. baumannii* populations (21, 22). Sequence types (STs) determined by MLST were then clustered into clonal complexes based on single-, double-, and triple-locus variants, corresponding to ICs. With the popularity of whole-genome sequencing (WGS) technology, core genome-based phylogenetic tree provides higher precision resolution than MLST (23).

Although there are increasing reports of *A. baumannii* causing disease in animals, there is still a lack of reports on whether this pathogen causes disease in poultry. A paucity of systematic analysis exists concerning the differences and interrelationships between *A. baumannii* isolated from animals and humans in terms of AMR and evolution. Consequently, there is an imperative for systematic studies of isolates from animals and humans to elucidate the relationship between *A. baumannii* transmission in animals and humans.

The aim of this investigation was to complement the data on *A. baumannii* causing disease in poultry and to elucidate the epidemiology and population structure of *A. baumannii* in animals by applying WGS to extract the MLST, core genome-based phylogenetic tree, ICs, capsular polysaccharide locus (KLs), and lipo-oligosaccharides outer core loci (OCLs). The isolates were grouped according to the number of AMR genes carried and then subjected to phylogenetic analysis in conjunction with isolates from humans in the same region. This was done to explore the lineage relationships of *A. baumannii* isolates from animals and humans and to provide early warning of potential public health safety threats.

## RESULTS

### Poultry *A. baumannii* exhibits characteristics of low AMR

The antibiotics used for testing adhered to the guidelines for antimicrobial susceptibility testing for *Acinetobacter*, as set out by the Clinical and Laboratory Standards Institute (CLSI) in M100 ED33-2023. Antibiotics with intrinsic resistance were excluded, such as ampicillin, amoxicillin, amoxicillin–clavulanate, aztreonam, ertapenem, trimethoprim, chloramphenicol, and fosfomycin. The results of antimicrobial susceptibility tests indicated that all the poultry isolates demonstrated susceptibility to 16 out of the 19 antibiotics examined (Table S1). In the susceptibility test for cefotaxime (CTX), three isolates (ZWAb045, ZWAb049, and ZWAb053) demonstrated susceptibility, while the remaining isolates exhibited intermediate susceptibility. In the susceptibility test for gentamicin (GM), only ZWAb053 exhibited intermediate susceptibility, and the rest of the isolates demonstrated susceptibility. In the susceptibility test for tetracycline (TE), ZWAb051 and ZWAb058 exhibited resistance; ZWAb046 and ZWAb067 demonstrated intermediate susceptibility; and the remaining isolates exhibited susceptibility.

**Fig 1:**
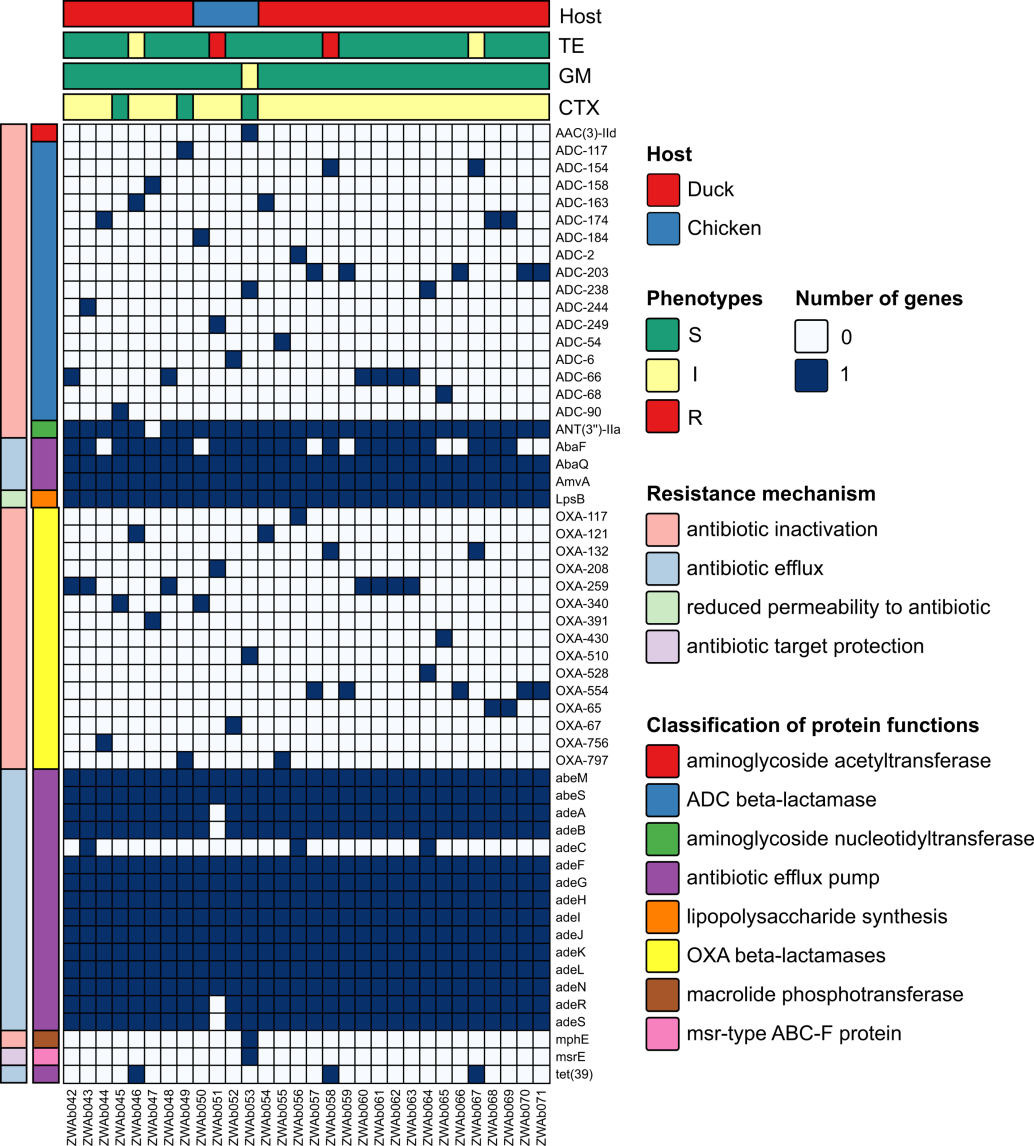
The present study analyzes the AMR genes and phenotypes of *A. baumannii* isolates from poultry. The isolates were fully susceptible to 16 of the 19 antibiotics tested and showed intermediate or resistant phenotypes to the remaining three: tetracycline (TE), gentamicin (GM), and cefotaxime (CTX). AMR genes were classified according to two schemes: (i) mechanism of resistance (four categories) and (ii) function of the encoded protein (eight categories).

A total of 30 genomes were subjected to a search for AMR genes, resulting in the identification of 55 genes (Fig. 1). These genes can be classified into four categories based on their mechanism of resistance, including antibiotic inactivation, antibiotic efflux, reduced permeability to antibiotics, and antibiotic target protection. Of these, the categories of antibiotic inactivation and antibiotic efflux contain 53 genes and represent the predominant resistance mechanisms. The encoded proteins can be categorized into eight distinct groups based on protein function, namely, aminoglycoside acetyltransferase, ADC β-lactamase, aminoglycoside nucleotidyltransferase, antibiotic efflux pump, lipopolysaccharide synthesis, OXA β-lactamases, macrolide phosphotransferase, and msr-type ABC-F protein. Among these genes, three classes collectively underlie the intrinsic resistance of poultry *A. baumannii* to certain antibiotics: *bla_ADC_* (*ampC*), *bla_OXA_* (class D β-lactamase), and *ade* (encoding an Ade-family resistance–nodulation–cell division-type efflux pump). The *bla_ADC_* and *bla_OXA_* genes exist in multiple allelic forms in *A. baumannii* and primarily encode β-lactamases. In the poultry *A. baumannii* isolates studied, each isolate harbored only one allelic variant of either *bla_ADC_* or *bla_OXA_*. Furthermore, expression of these intrinsic resistance genes often requires insertion of ISAba1 upstream to enhance promoter activity and confer phenotypic resistance. However, ISAba1 was not detected in the poultry embryo-sourced *A. baumannii* isolates, indicating that the observed changes in antibiotic resistance are not associated with the insertion sequence ISAba1. The Ade efflux system primarily mediates antimicrobial efflux but typically requires overexpression to exert a functional resistance phenotype. Notably, these intrinsic resistance mechanisms were not reflected in the antibiotic susceptibility testing results for the poultry *A. baumannii* isolates.

Comparative analysis of predicted AMR genes and antimicrobial susceptibility phenotypes revealed that tetracycline resistance in these isolates was generally associated with the presence of the tet (39) resistance gene, as strains harboring this gene exhibited intermediate or resistant phenotypes to tetracycline. However, isolate ZWAb051, despite lacking the *tet*(39) gene, also displayed tetracycline resistance, suggesting the existence of alternative tetracycline resistance genes or mechanisms. Furthermore, the observed variations in resistance to gentamicin and cefotaxime could not be attributed to any known corresponding resistance genes in the genomes, implying that other factors, such as efflux pump overexpression or chromosomal target mutations, may be responsible. Regarding host origin, no significant differences were observed in the resistance gene carriage profiles between chicken-derived and duck-derived isolates.

### Identification of new loci for KL and OCL in clones isolated from poultry

**Fig 2:**
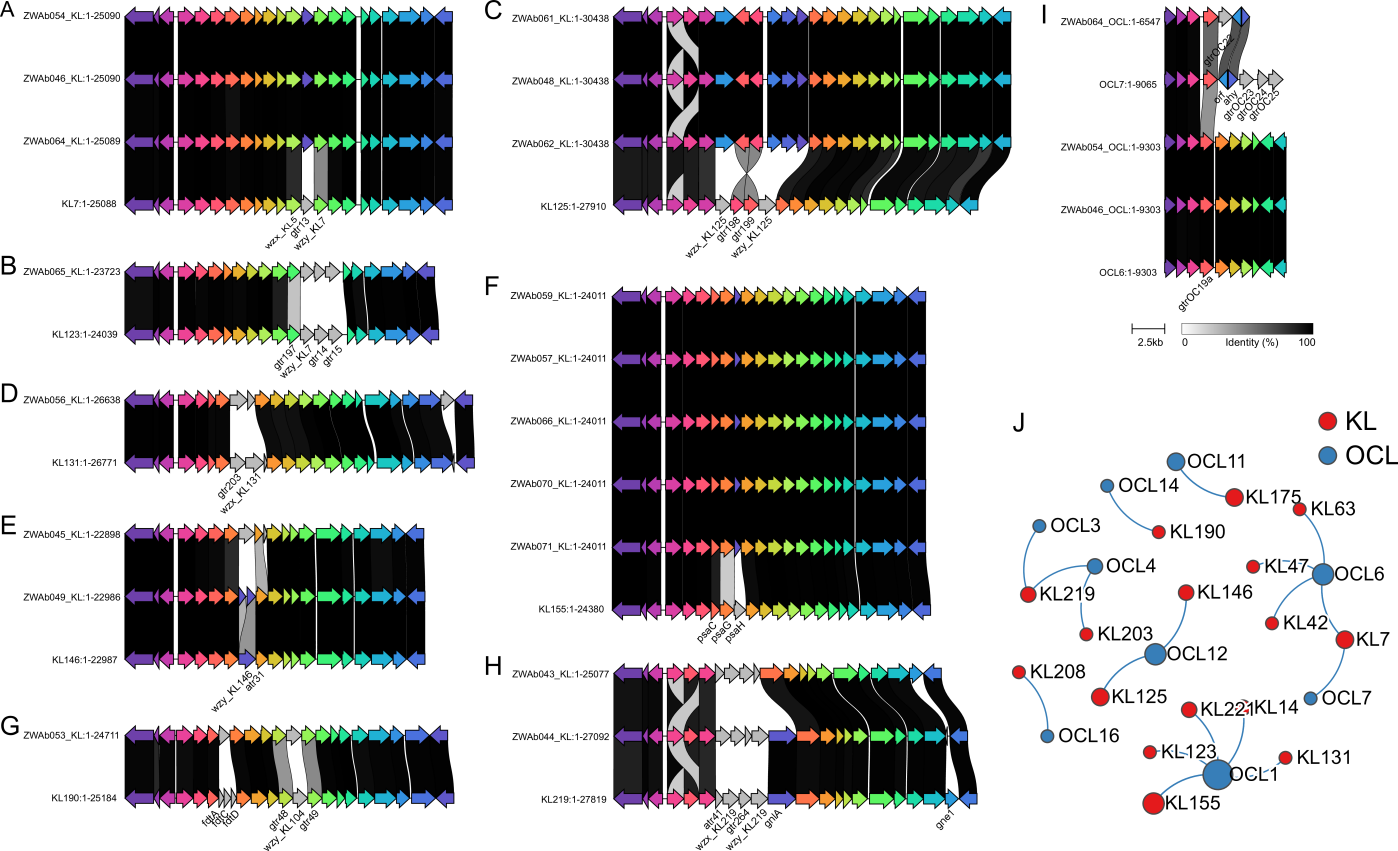
KL and OCL typing of *A. baumannii* isolates from poultry. (**A–H**) Comparison with the Kaptive v3.0 KL reference database identified nine novel KL types, likely generated through horizontal gene transfer. (**I**) A putative novel OCL type (designated ZWAb064_OCL). (**J**) Association between KL and OCL types across isolates, with circle size proportional to the number of isolates sharing each KL_OCL combination.

By alignment with the KL and OCL gene clusters in the Kaptive v3 database, nine new KL gene clusters and one new OCL gene cluster were identified, including KL7-like, KL123-like, KL125-like, KL131-like, KL146-like, KL155-like, KL190-like, and two new KL219-like. These new KL types arise mainly from horizontal gene transfer of the *wzx*, *wz*y, and *gtr* genes within their gene clusters (Fig. 2A through H). A novel OCL7-like gene cluster was identified (Fig. 2I), and significant mutations were observed in the *gtrOC22* (70%), *orf* (76%), and *ahy* (92%) genes of the OCL7-like gene cluster compared with the OCL7 gene cluster. An additional gene was inserted between *gtrOC22* and *orf* in the OCL7-like gene cluster. Furthermore, analysis of the data indicates that ZWAb046, ZWAb054, and ZWAb064 belong to the same KL7 type, but their OCL gene clusters have been altered, resulting in different OCL types. The increasing diversity of KL and OCL types, as well as the increasing number of KL-OCL combinations, determines the serotype diversity of *A. baumannii*. In the genomes of these 30 isolates, 16 KL types and 9 OCL types were predicted, and combining these two types resulted in 18 KL-OCL types (Fig. 2J). The diversity of the CPS and LOS indicates the genetic diversity of *A. baumannii* in poultry.

### Characteristics of virulence genes in poultry *A. baumannii*

**Fig 3:**
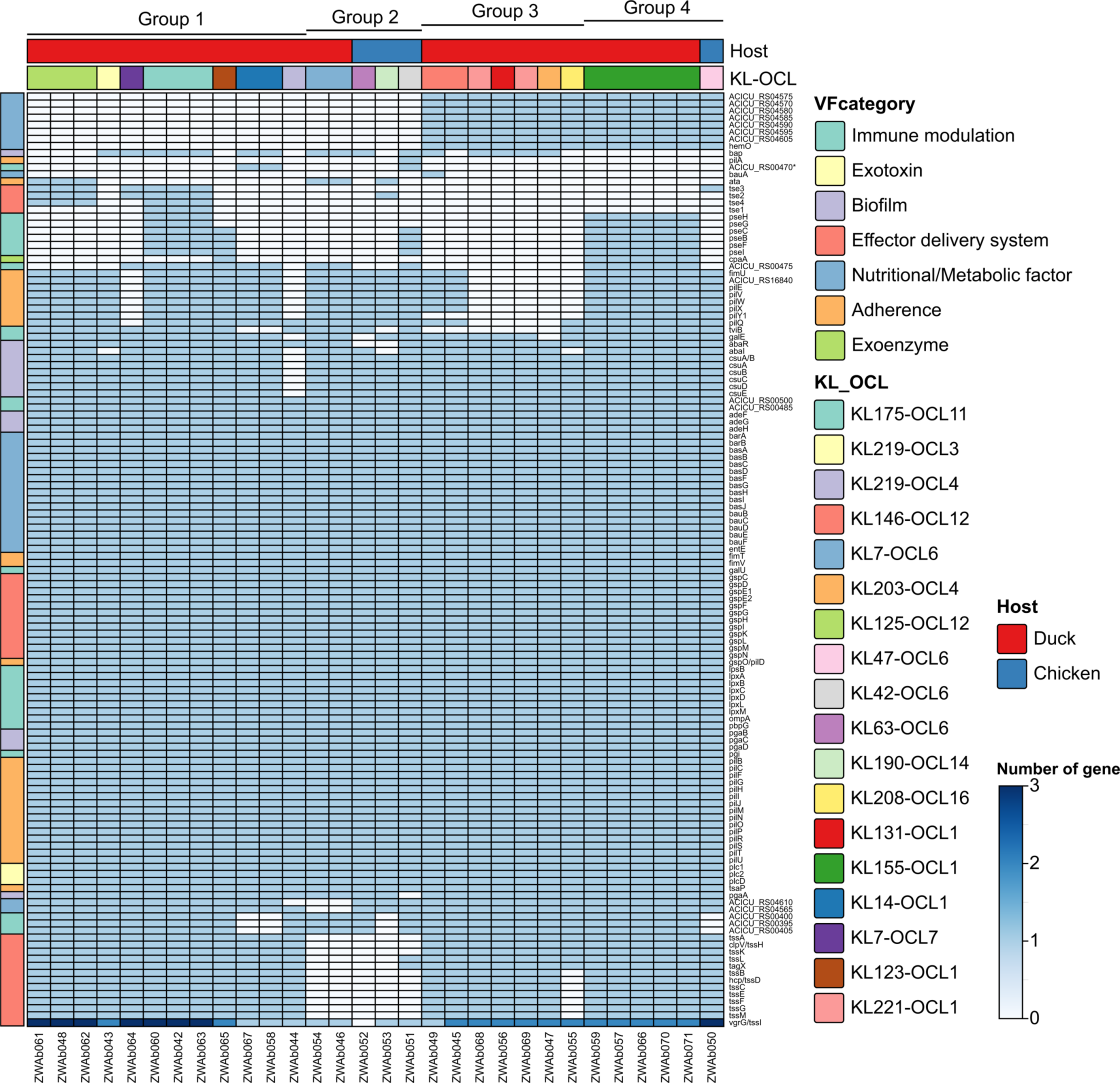
Virulence gene analysis of *A. baumannii* isolates from poultry. Isolates were grouped into four groups based on virulence gene profiles. Virulence-associated genes were categorized into seven functional classes, including immune modulation, exotoxin, biofilm, effector delivery system, nutritional/metabolic factor, adherence, and exoenzyme.

An investigation was conducted into the virulence genes of 30 *A. baumannii* isolates from poultry (Fig. 3). The results of this investigation demonstrated that the virulence genes carried by different isolates differed significantly from each other. A total of seven types of virulence genes were retrieved in these isolates, including immune modulation, exotoxin, biofilm, effector delivery system, nutritional/metabolic factor, adherence, and exoenzyme. Among these, the five categories of virulence-associated genes, including immune modulation, biofilm, effector delivery systems, nutritional/metabolic factors, and adherence, are the primary contributors to the virulence of poultry *A. baumannii*. Poultry *A. baumannii* lacks virulence genes associated with exotoxins and exoenzymes, consistent with the typical pathogenicity pattern of *Acinetobacter* species, which rely not on potent exotoxins but on biofilm formation, immune modulation, and adhesion to establish chronic or opportunistic infections. Based on the distribution of virulence genes, the 30 *A. baumannii* isolates can be grouped into four clusters. The isolates in groups 1 and 2 lack predominantly certain nutritional/metabolic factor and immune modulation-related virulence genes. Additionally, Group two isolates lack a significant proportion of effector delivery system–related virulence genes. Groups 3 and 4 primarily lack certain effector delivery system–related virulence genes, and Group three also lacks certain virulence genes associated with immune modulation and adherence. The compositional differences in virulence genes among the groups indicate variations in environmental adaptability, nutrient acquisition capacity, and pathogenic potential of these isolates. Each group contains multiple KL_OCL-type isolates, and isolates of the same KL_OCL type exhibit identical virulence gene compositions. With respect to host species, no significant differences in virulence gene composition were observed between chickens and ducks. The observed variation in virulence genes further underscores the genomic diversity of these isolates. However, due to the limited number of isolates, these characteristics may require further validation with additional data.

### Characteristics of plasmids and prophage in poultry *A. baumannii*

**Fig 4:**
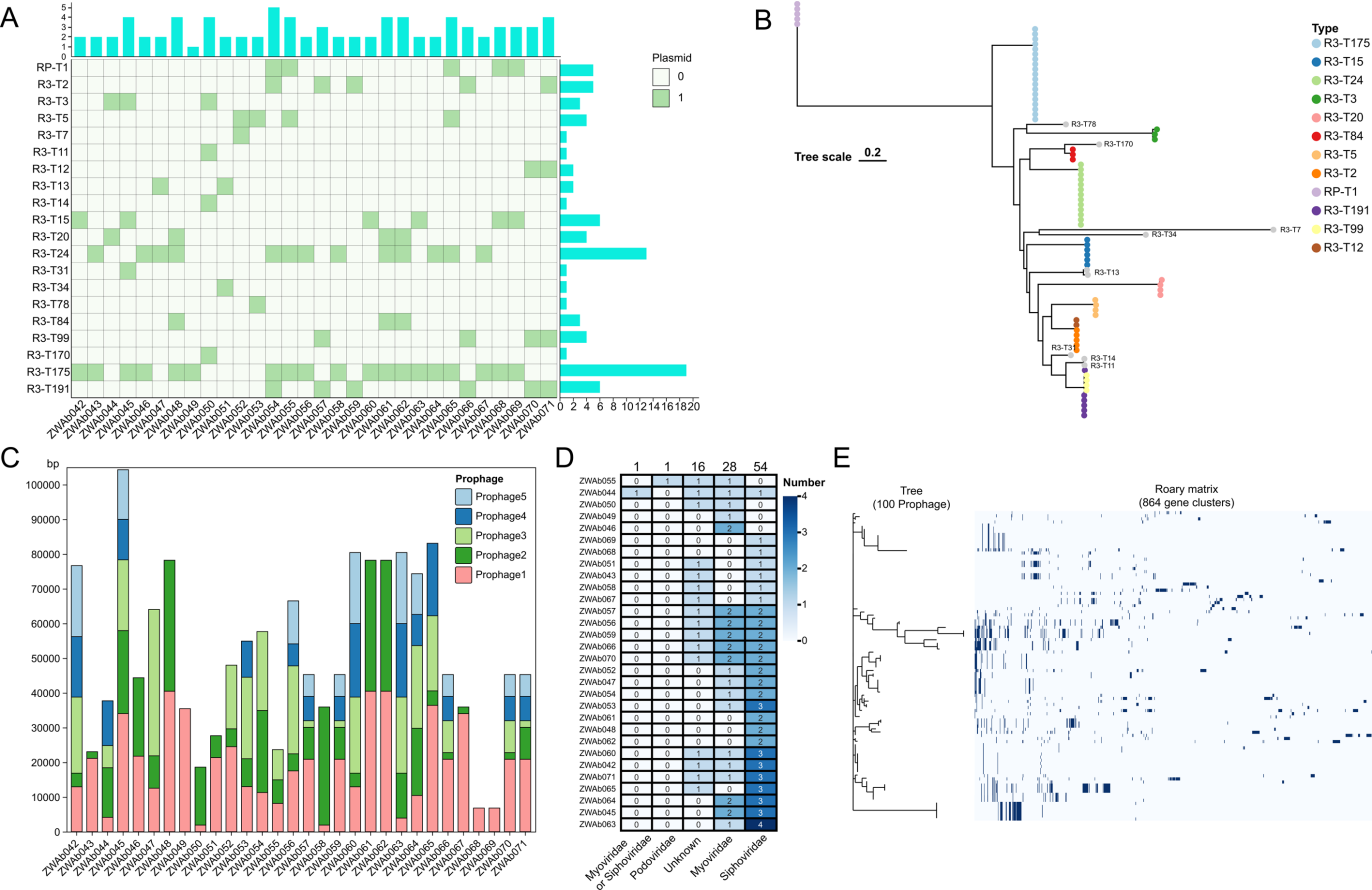
Plasmid and prophage analyses of *A. baumannii* isolated from poultry. (**A**) Distribution of different types of plasmids in poultry embryo-sourced *A. baumannii* isolates. (**B**) Maximum-likelihood phylogenetic relationship of replication initiation Rep sequences in plasmids. (**C**) Size and distribution of prophages in *A. baumannii* isolates. (**D**) Morphological classification of prophages in different isolates, predominantly myoviruses and siphoviruses. (**E**) Phylogenetic analysis of prophages. The left panel shows a tree based on gene presence/absence profiles; the right panel displays a binary matrix of all prophage-encoded genes across isolates.

A total of 83 genes encoding replication initiation (Rep) proteins were identified among 30 *A. baumannii* isolates recovered from avian embryos, indicating that these isolates harbor at least 83 plasmids (Fig. 4A). Based on Rep protein classification, these plasmids fall into two major groups comprising 20 distinct types, with Rep_3-type plasmids being overwhelmingly predominant. Previous work has established that Rep_3 plasmids are the most frequently carried replicons in *A. baumannii* and that subtypes R3-T1 and R3-T2 dominate among human clinical isolates (24–26). In contrast, among the avian embryo-derived isolates examined here, R3-T1 and R3-T2 subtypes were not the most prevalent, revealing a notable divergence in plasmid population structure from that of human-associated strains. Instead, the R3-T175 subtype was the most abundant plasmid in the avian embryo isolates, accounting for approximately 63% of the population, and it harbored the tetracycline resistance determinants *tet(A)* and *tet(B)*. With the sole exception of isolate ZWAb049, every strain carried at least two plasmids, and isolate ZWAb054 exhibited the highest plasmid burden. RP-T1-type plasmids have been implicated in the horizontal dissemination of the carbapenem resistance gene *bla_OXA-23_* and the amikacin resistance gene *aphA6* (24). These plasmids contain intact conjugation-related gene clusters, and some have been experimentally demonstrated to be conjugative (27, 28). In the present collection of avian embryo-derived *A. baumannii* isolates, RP-T1 plasmids were detected in only five strains, and notably, neither *bla_OXA-23_* nor *aphA6* was identified in any of these isolates. Phylogenetic analysis of the Rep proteins revealed that Rep_3 homologs can be further resolved into distinct subtypes, reflecting considerable sequence diversity within their coding genes (Fig. 4B). Moreover, within a given subtype, the rep genes carried by different isolates exhibited further intrasubtype diversification, as exemplified by the additional branching observed for subtypes R3-T3, R3-T13, and R3-T191.

A total of 100 prophage DNA sequences were identified in these isolates (Fig. 4C). Each isolate carried a maximum of five prophage DNA sequences, a minimum of one, and a median of three. A global estimate of prophages in *A. baumannii* populations indicated that the median is three prophages per bacterial isolate for humans; the median for both animal and plant isolates is slightly over 2 (29). The median number of prophages in our poultry isolates was comparable to that in human isolates, but this similarity may stem from differences in identification methods, regional biases, or host-specific biases. According to the classical phage morphological classification, the identified prophages could be classified into three groups: Siphoviridae (*n* = 54), Myoviridae (*n* = 28), and Podoviridae (*n* = 1). However, the classification of some prophages was undetermined due to the lack of taxonomic markers (*n* = 17). This finding indicates that prophages of Siphoviridae and Myoviridae morphology are predominant among all prophages of *A. baumannii* isolated from poultry (Fig. 4D). Phylogenetic trees and gene distribution matrices, constructed on the basis of gene presence and absence, indicate the complex diversity of prophage element insertions in these isolates (Fig. 4E). No known virulence factors and AMR genes were identified in any of the prophages. The diversity of plasmids and prophages is one of the factors contributing to the diversity of *A. baumannii* isolated from poultry.

### Genetic diversity of *A. baumannii* clones isolated from poultry

**Fig 5:**
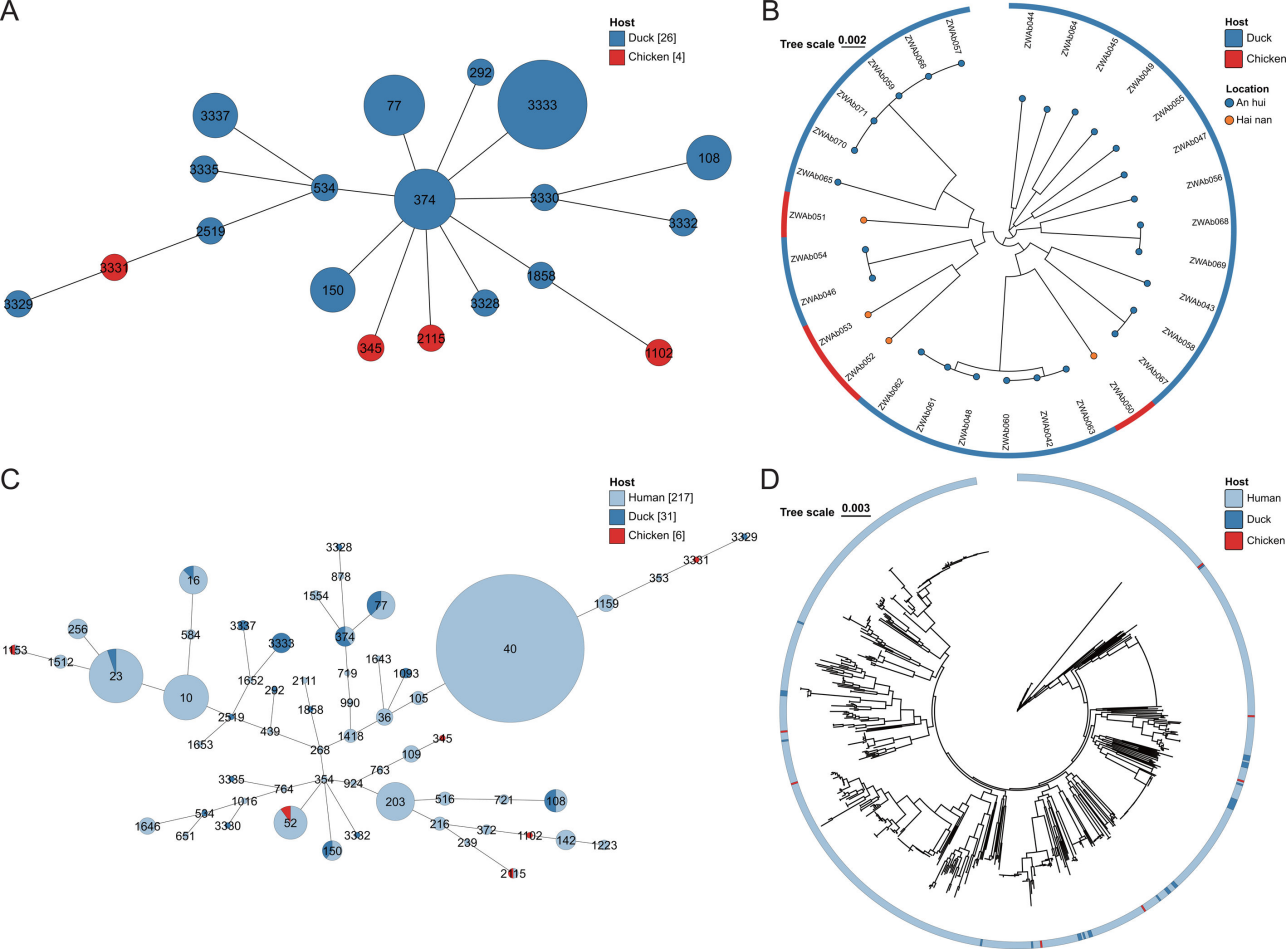
Comparative genomic analyzes of *A. baumannii* isolated from poultry and humans. (**A**) MLST profiling of *A. baumannii* isolates from poultry. (**B**) Phylogenetic analyzes of *A. baumannii* isolates from poultry. (**C**) MLST profiling of *A. baumannii* isolates from humans, chickens, and ducks in China. (**D**) Phylogenetic analyzes of *A. baumannii* isolates from humans, chickens, and ducks in China.

To investigate the phylogenetic relationships among the poultry isolates in this study, ST typing revealed that the 30 isolates could be assigned to 19 STs, of which 10 were previously identified, including ST77, ST108, ST150, ST292, ST345, ST374, ST534, ST1102, ST1858, and ST2115, and the remaining 9 represented novel STs. These novel STs were submitted to PubMLST and assigned specific designations, including ST2519, ST3328, ST3329, ST3330, ST3331, ST3332, ST3333, ST3335, and ST3337. In these STs, ST374 was located at the central node of the minimum spanning tree, indicating that the other STs likely descended from this lineage. The minimum spanning tree analysis further revealed a degree of evolutionary relatedness between *A. baumannii* isolates from chickens and ducks (Fig. 5A). Specifically, chicken *A. baumannii* of ST3331 occupies an intermediate position in the evolutionary pathway between duck ST2519 and ST3329. Chicken *A. baumannii* of ST1102 appears to have evolved from duck *A. baumannii* of ST1858, while chicken *A. baumannii* of ST345 and ST2115 appear to have evolved from duck *A. baumannii* of ST374. A higher-resolution core-genome phylogenetic tree supported these findings (Fig. 5B). Notably, isolates from chickens and ducks did not form distinct monophyletic clades, suggesting no apparent host specificity of the pathogen between these two avian hosts.

To investigate the relationship between poultry and human isolates, we performed phylogenetic analyses using these isolates and a total of 785 *A. baumannii* genomes from China in NCBI’s GenBank database, with host markers indicating duck (*n* = 5), chicken (*n* = 3), and human (*n* = 747). ST2 *A. baumannii* was the most prevalent clone among human isolates in China; however, it was not detected among the poultry isolates in this study and was therefore excluded from the analysis. To analyze the evolutionary relationship between poultry and human *A. baumannii*, a minimum spanning tree based on STs and a higher-resolution core-genome phylogenetic tree were constructed. Among 785 *A. baumannii* genomes, 157 known STs were identified, with 144 genomes remaining unassigned to an ST. To facilitate observation of the evolutionary relationship between poultry and human *A. baumannii*, branches unrelated to poultry isolates were pruned from the minimum spanning tree, leaving 217 human *A. baumannii* genomes for evolutionary analysis (Fig. 5C). Among the numerous STs, only a few are shared between human and poultry *A. baumannii*, including ST16, ST23, ST52, ST77, ST108, ST150, ST374, ST1153, and ST2115. These STs represent a very small proportion of human isolates and correspond to rare infection cases, indicating that poultry isolates have the potential to infect humans, albeit with likely lower pathogenicity. A higher-resolution core genome-based phylogenetic tree supports this finding (Fig. 5D). Poultry isolates did not form a distinct clade in the phylogenetic tree, suggesting no apparent host specificity. Instead, poultry isolates are interspersed within multiple branches of the human isolates, indicating a potential transmission risk between poultry and humans. However, the relatively distant evolutionary relationships between poultry and human isolates within the same branch suggest that this risk may be very low. Notably, when AMR profiles are considered, poultry *A. baumannii* exhibits widespread susceptibility to CLSI-recommended antibiotics, in stark contrast to the multi-drug resistance commonly observed in human clinical isolates. Nevertheless, given the capacity of *A. baumannii* for rapid acquisition of resistance determinants, continued vigilance regarding this potential transmission pathway remains warranted.

### Population structure of *A. baumannii* clones isolated from animals worldwide

**Fig 6:**
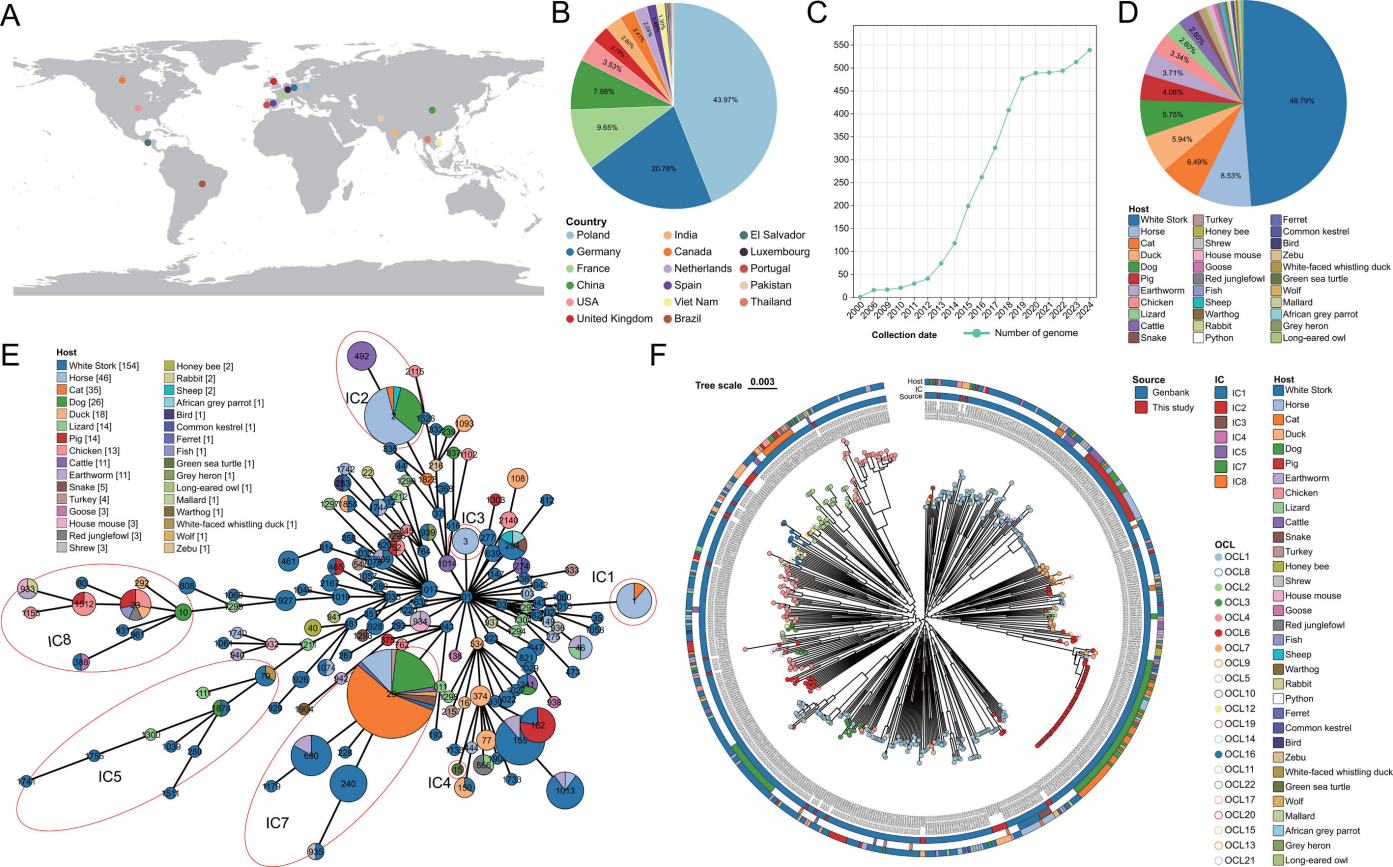
The global population structure of *A. baumannii* isolated from animals. (**A**) Geographical distribution of animal-derived *A. baumannii* isolates. Base map data: Baidu Maps (https://map.baidu.com). Map generated using Chiplot Online (https://www.chiplot.online/map_plot.html). (**B**) Proportion of *A. baumannii* isolates from animals across 17 countries. (**C**) Temporal trends in the number of *A. baumannii* isolates from animals over the past 20 years. (**D**) Proportion of *A. baumannii* isolates from different animal hosts. (**E**) Global MLST analyses of *A. baumannii* isolates from animals. (**F**) A phylogenetic tree based on the core genome of *A. baumannii* isolates from animals worldwide.

A total of 509 *A. baumannii* isolates from animals were retrieved from the NCBI GenBank database and used in conjunction with the 30 isolates from this study to analyze the global distribution of the pathogen in animals. As of 2025, *A. baumannii* has been isolated from animals in 17 countries worldwide, including Poland, Germany, France, China, the United States, the United Kingdom, India, Canada, the Netherlands, Spain, Vietnam, Brazil, El Salvador, Luxembourg, Portugal, Pakistan, and Thailand (Fig. 6A). Poland was the country with the highest number of *A. baumannii* isolates from animals, accounting for approximately 43.97% of the total, with the majority of these being isolated from the wild species, the white stork (Fig. 6B). Germany demonstrated a percentage of approximately 20.78%. In France and China, a significant number of animal *A. baumannii* isolates were obtained, accounting for 9.65% and 7.98%, respectively. The study showed that the first animal *A. baumannii* was isolated from cats in Germany in 2000 (Fig. 6C). After 2009, there was a marked increase in the number of animal isolates, which continued until 2020. Research on animal *A. baumannii* was hampered in 2020–2022, probably due to the COVID-19 epidemic. After the epidemic, research on animal *A. baumannii* resumed. To date, *A. baumannii* has been isolated worldwide from more than 33 species of animals (Fig. 6D), such as the white stork (48.79%), horse (8.53%), cat (6.49%), duck (5.94%), and dog (5.57%). The available data suggest that *A. baumannii* has become an important pathogen in animals (9, 11, 12, 30, 31).

Based on the seven-loci MLST analysis in the Pasteur scheme, a total of 396 genomes were assigned an ST among 539 animal isolates, while the remaining 143 genomes could not be assigned an ST. A total of 170 STs were identified in these isolates, of which eight—ST25, ST2, ST155, ST690, ST1013, ST240, ST1, and ST162—were the major STs. In accordance with the definition of a clonal complex as a determination criterion, each isolate was assigned to one of the eight established ICs. Isolates from animals were assigned to seven of the eight ICs, with the exception of IC6. The distribution of ICs is shown in Fig. 6E. IC7 is the largest clonal lineage and includes the largest number of hosts. It is also important to note that IC2, IC5, and IC8 are significant clonal lineages among animal isolates. However, many isolates are unique clones that differ from these eight ICs. Alarmingly, among the ST25 clones within the predominant IC7 lineages, cats, dogs, and horses are the predominantly infected hosts. It is evident that other significant ICs associated with human infections, including IC1, IC2, and IC3, are in a similar situation. This finding suggests that infections in these hosts may exhibit a greater degree of correlation with human infections than with those observed in other hosts.

A phylogenetic tree based on core-genome alignment demonstrated that the 30 isolates in this study were dispersed throughout the evolutionary tree without regional or host specificity (Fig. 6F). According to the clustering of ICs, two major ICs are clearly clustered in the tree, including IC2 and IC7. A high degree of conservation was observed in the OCL types of both ICs. These animal isolates exhibit exceptionally high conservation within the ICs, whereas those outside the ICs show highly divergent evolutionary relationships. A similar pattern is observed with respect to host associations: apart from horse, cat, and dog isolates, which cluster within a few human-associated ICs, other animal isolates show no apparent host specificity. Isolates from animals were characterized by distinct clustering in terms of OCL typing. In cluster 1 (10 o’clock), OCL2, OCL4, OCL6, and OCL16 were predominantly observed, while in cluster 3 (4 o’clock), OCL6 and OCL7 were the dominant types. By contrast, in cluster 2 (6 o’clock) and cluster 4 (1 o’clock), OCL1 was the most prominent. This suggests that the evolution of OCL gene clusters is relatively more conserved despite the high diversity of animal isolates and can be used as an evolutionary marker.

### Zoonotic potential of *A. baumannii* clones isolated from animals

To research the potential of cross-host transmission of *A. baumannii*, we conducted phylogenetic analysis of the animal isolates in comparison to the human isolates. In consideration of the capacity of *A. baumannii* to acquire AMR, we classified the animal isolates into two primary categories based on the number of AMR genes carried, including a low-AMR group (isolates with low AMR profiles, *n* ≤ 20) and a high-AMR group (isolates with high AMR profiles, *n* ≥ 21). A categorization of the isolates according to their hosts revealed that livestock, poultry, and various wildlife were predominant in the low-AMR group, whereas companion animals such as horses, cats, and dogs were predominant in the high-AMR group. Furthermore, human isolates were selected according to the geographical location of the hosts.

**Fig 7:**
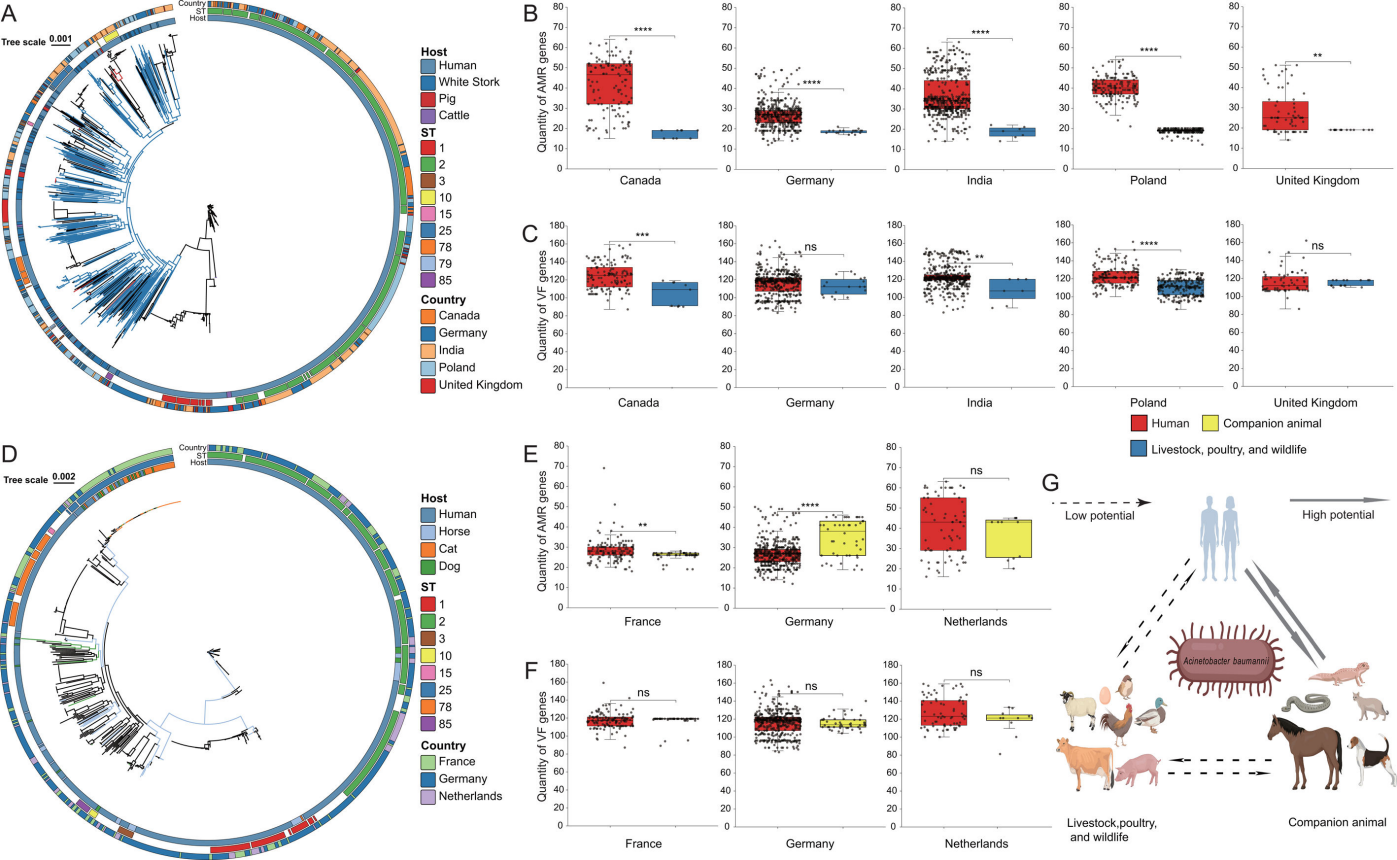
An investigation into the MLST, phylogenetic analysis, antibiotic resistance, and virulence genes of *A. baumannii* isolates from animals and humans in the same region. (**A**) Phylogenetic tree constructed based on the core genome of animal-associated *A. baumannii* isolates with low AMR profiles and human isolates from the same region. (**B**) Comparative analysis of AMR genes between animal-associated *A. baumannii* isolates with low AMR profiles and human isolates from the same region. (**C**) Comparative analysis of virulence genes between animal-associated *A. baumannii* isolates with low AMR profiles and human isolates from the same region. (**D**) Phylogenetic tree based on the core genome of animal-associated *A. baumannii* isolates with high AMR profiles and human isolates from the same region. (**E**) Comparative analysis of AMR genes between animal-associated *A. baumannii* isolates with high AMR profiles and human isolates from the same region. (**F**) Comparative analysis of virulence genes between animal-associated *A. baumannii* isolates with high AMR profiles and human isolates from the same region. (**G**) Inferred transmission potential of *Acinetobacter baumannii* among humans, companion animals, livestock, poultry, and wildlife, created with biogdp.com. ns, non-significant (*P*＞0.05); **, *P* ≤ 0.01; ***, *P* ≤ 0.001; ****, *P* ≤ 0.0001. The colors of the branches in the phylogenetic tree represent the host classification, but “human” is by default marked in the original black color on the phylogenetic tree to facilitate distinction.

For the low-AMR group, the top three hosts were selected based on the number of isolates, with a total of 286 isolates from white storks (*n* = 255), pigs (*n* = 18), and cattle (*n* = 13). These isolates were sourced from the following countries: India, Canada, the United Kingdom, Germany, and Poland. Therefore, for human isolates, a total of 1,263 *A. baumannii* isolates in these five countries were selected for analysis. A core genome-based phylogenetic tree was constructed using these isolates, revealing no significant regional specificity among the isolates (Fig. 7A). These isolates did not belong to the nine major ST lineages highly associated with humans and exhibited more distant relationships along human-associated evolutionary branches. Analysis of their virulence and resistance genes further supported these conclusions (Fig. 7B and C). Comparisons between animal isolates and human isolates by country revealed that animal isolates carried significantly fewer resistance genes than human isolates from the same regions. Regarding virulence genes, animal isolates from all regions except Germany and the United Kingdom also carried significantly fewer virulence genes than human isolates. Notably, human isolates from Germany and the United Kingdom carried fewer virulence genes than those from the other three regions, with a median of fewer than 120.

For the high-AMR group, the top three hosts were selected based on the number of isolates, with a total of 106 isolates included in the study. These hosts included isolates from horses (*n* = 46), cats (*n* = 33), and dogs (*n* = 27). The majority of the isolates from these three sources were from European countries, specifically France, Germany, and the Netherlands. Therefore, for human isolates, a total of 654 *A. baumannii* isolates from these three countries were selected for analysis. A core genome-based phylogenetic tree was constructed using these isolates, revealing no significant regional specificity among the isolates (Fig. 7D). Most companion animal *A. baumannii* isolates clustered within a few lineages on the tree and exhibited very close relationships to human isolates. These isolates belonged to lineages highly associated with humans, such as ST1, ST2, ST3, ST10, and ST25. Only a few companion animal isolates were located in other lineages (at the 7–9 o’clock positions). Furthermore, isolates within other lineages exhibited more distant relationships to human isolates. Analysis of virulence and resistance genes supported these conclusions (Fig. 7E and F). Comparisons between companion animal and human isolates by country revealed that companion animal *A. baumannii* carried resistance and virulence genes similar to those found in humans.

Finally, we analyzed the types of resistance genes carried by these four populations. Results indicate that companion animal isolates carried 68 resistance genes, sharing 65 with human isolates from the same region. Only three resistance genes were unique: *bla_OXA-121_* (*n* = 4), *bla_OXA-339_* (*n* = 4), and *bla_OXA-413_* (*n* = 4). The livestock, poultry, and wildlife isolates collectively carried 111 resistance genes, sharing 80 with human isolates from the same region and possessing 31 unique resistance genes. This indicates that companion animal isolates are highly similar to human isolates and do not exhibit greater uniqueness (Fig. S1). Furthermore, we compared the resistance gene profiles between isolates from companion animals and those from livestock, poultry, and wildlife. We found that companion animal isolates carried 20 resistance genes not detected in the livestock, poultry, and wildlife isolates, including multiple carbapenem resistance genes such as *bla_NDM-1_* (*n* = 2) and *bla_OXA-23_* (*n* = 100). As the “last line of defense” against multi-drug-resistant bacterial infections in humans, carbapenem antibiotics are strictly prohibited in livestock farming. The detection of these carbapenem resistance genes further supports the high genetic similarity between companion animal and human isolates. Differences in virulence gene repertoires among the groups also corroborate this finding, albeit less markedly than the differences observed in resistance genes (Fig. S2). This is because *A. baumannii* is notorious not for its virulence but for its antibiotic resistance, employing a “persistence and resistance” strategy in pathogenesis. The results of average nucleotide identity (ANI) also demonstrate that companion animal isolates exhibit a high degree of similarity to human isolates compared to isolates from livestock, poultry, and wildlife (Fig. S3 to S10). The ANI values between companion animal isolates and human isolates are mostly above 99%, even reaching over 99.5% (Fig. S3 to S5). In contrast, the ANI values between human isolates and isolates from livestock, poultry, and wildlife are generally below 99%, ranging between 97% and 98% (Fig. S6 to S10). Only a small proportion of isolates can achieve ANI values between 98% and 99%. In particular, the white stork isolates form a distinct population separate from the human isolates (Fig. S9).

Therefore, the integration of the results suggests a higher potential of intertransmission between companion animals and humans. This elevated potential may be related to the close contact of companion animals with humans. Conversely, domestic animals, poultry, and wildlife exhibit a low potential of *A. baumannii* transmission due to their low contact with humans and companion animals.

## DISCUSSION

*A. baumannii*, an emerging zoonotic pathogen, poses a significant threat to animal and human life and health (32–35). However, the population structure of the pathogen in animals and the risk of transmission between humans and animals have not been systematically studied.

The present study constitutes an investigation into the pathogenicity of *A. baumannii* in poultry, alongside the AMR characteristics and genetic diversity of isolates. According to data from the China Antimicrobial Resistance Surveillance System (https://www.carss.cn/), the resistance rate of *A. baumannii* clinical isolates to carbapenem antibiotics is approximately 75%. However, the findings of this study demonstrated that *A. baumannii*, which was isolated from the embryos of poultry that failed to complete hatching, exhibited a high degree of susceptibility to the antibiotics recommended by CLSI. The low AMR of the pathogen isolated from poultry may be attributable to the strict antiantibiotic restriction and ban policy implemented by the Chinese government in the poultry industry, particularly in laying hens. Interestingly, the two plasmids in the isolates isolated in this study had the highest homology with the plasmids of *A. baumannii* Y03 (SAMN42799853) (36) and HNNY86G (SAMN38345664) that have been reported to have been isolated from morbid chickens in Shandong, China, and from throat swabs of healthy chickens in Henan, China. This also suggests that *A. baumannii* isolated from poultry in different regions may have a very close genetic correlation. However, further research is required to ascertain the pathogenicity of *A. baumannii* in poultry, as the current literature is dominated by reports of *A. baumannii* isolates from tracheal and rectal swab samples from healthy poultry (33, 37, 38). Moreover, 16 KL and 9 OCL types were identified in the isolates in this study. Notably, nine KL gene clusters and one OCL gene cluster were not identical to the known gene clusters, which were identified for the first time in this study.

Secondly, the population structure of *A. baumannii* in animals was analyzed systematically for the first time. Among these IC lineages, IC2, IC5, IC7, and IC8 were the most dominant in companion animals. In particular, isolates of type ST25 in IC7 have been reported in the past to be the predominant clonal lineage of *A. baumannii* infecting cats and dogs in Germany and France (35, 39). In the present study, we found that isolates of the ST25 clone were isolated in nine species of animals with a very broad spectrum of infection. In addition, ST1, ST2, ST25, and ST79 of the four major international clonal lineages detected in companion animals are the most dominant of the publicly available genome sequences, accounting for approximately 71% of all sequenced genomes or more (40). ST2 is by far the most globally prevalent clonal lineage in humans and is also strongly associated with infections in companion animals (41–43).

The IC lineage is a broad concept encompassing numerous distinct ST branches (44–46). An IC is typically composed of a primary ST along with other STs that share high evolutionary relatedness with it. This primary ST exhibits a strong association with humans. However, using only seven housekeeping genes for ST analysis, out of *A. baumannii*’s approximately 3,000–4,000 genes, presents a significant limitation: markedly insufficient resolution. Core genome-based cgMLST offers substantially higher resolution than conventional MLST (46). Therefore, we extracted the core genome of *A. baumannii* and constructed a phylogenetic tree based on core-genome alignment. Within this tree, we observe that ST492 cattle isolates within the IC2 lineage share an evolutionary branch with ST2 isolates, whereas ST429 has clearly diverged into a separate branch. In IC7, ST25 consists almost entirely of companion animal isolates, while the remaining STs in this lineage are predominantly derived from white storks. The tree reveals that the white stork isolates and the ST25 isolates have completely diverged into two distinct clades. IC8 is primarily composed of isolates from domestic animals, poultry, and wildlife. ST10, designated as the putative ancestral node of this lineage, contains only two dog isolates. The remaining dog isolates are primarily concentrated within the IC2 and IC7 lineages. Although other animal isolates within IC8 share the same lineage designation, they have gradually diverged evolutionarily from the primary clone, ST10. Furthermore, the IC8 lineage exhibits significantly lower prevalence in humans compared to other ICs, showing only limited local circulation in Europe relative to other globally disseminated clones (45). Within the tree, we also observe that while some IC8 isolates cluster on the same major branch, others are scattered across distantly related branches, indicating substantial phylogenetic heterogeneity within this lineage.

Research focusing on the transmission of *A. baumannii* associated with wildlife and poultry has proposed and validated birds as potential reservoirs for this pathogen (33). However, studies indicate that while rare individual isolates from poultry and white storks have been linked to human clinical isolates in China or the United States, the vast majority of isolates belong to new, diverse lineages unrelated to human clinical isolates. Furthermore, studies indicate that the virulence of these wildlife and poultry associated isolates is comparable to that of human clinical isolates in *Galleria mellonella* infection models. It is noteworthy that *A. baumannii* lacks clearly identified toxins in human clinical infections and is currently widely recognized as employing a persistence and resistance strategy. Regarding antimicrobial resistance, apart from inherent resistance to ampicillin and cephalosporins, these isolates exhibit no resistance to other common antibiotics. Findings from studies on *A. baumannii* isolates from pigs and cattle further support these results. Isolates from cattle and pigs represent a new clonal lineage distinct from major ICs, exhibiting fewer resistance and virulence genes compared to human isolates (15, 47, 48). Collectively, these data demonstrate that *A. baumannii* isolates from companion animals pose a greater potential threat to humans than those from wildlife, livestock, and poultry.

*A. baumannii* has been isolated from many animals, both pathogenic and non-pathogenic. Animals that suffer from the infection include mainly horses (12), cats (9, 49), dogs (50, 51), sheep (13, 14), minks (52), and falcons (53). *A. baumannii* was detected in such animals and linked to signs of infection in the animals. As for the non-diseased animals, mainly pigs (15), cattle (15, 54), chickens (37, 38), ducks (55), geese (33, 38), and wild birds (33), the sampling sites focused on throat swabs, anal swabs, feces, and samples from the farming environment (47). Such tests are more for enriching studies on the epidemiology of *A. baumannii* in animals and on the spread of AMR genes. In addition, relevant research studies have been conducted on meat foods (56–58), including pork, beef, and chicken. This further raises the issue of foodborne safety of this pathogen. In this study, data analysis from multiple perspectives, including AMR genes, virulence genes, MLST, and core genome-based phylogenetic trees, revealed that *A. baumannii* isolated from a companion animal showed a higher potential of transmission between companion animals and humans than wildlife, livestock, and poultry. Reasons for this phenomenon may include high exposure, high-dose antibiotic use in veterinary hospitals, and the evolution of host adaptations in some clonal lineages. Notably, extensive exposure of companion animals to humans may represent a significant risk factor for the transmission of this pathogen. Furthermore, *A. baumannii* is known to be a serious nosocomial pathogen that primarily affects middle-aged and elderly populations (59). Companion animals could potentially serve as intermediate vectors in the transmission of this pathogen, thereby posing additional risks to these vulnerable groups, particularly in regions such as China, countries in Western Europe, the United States, and Japan, where population aging is becoming increasingly pronounced.

### Conclusions

In summary, this study fills a research gap by finding that *A. baumannii* can cause mortality in incompletely hatched poultry embryos. Antimicrobial susceptibility testing revealed that *A. baumannii* isolates from dead poultry embryos exhibited widespread susceptibility to antibiotics recommended by CLSI. ST typing and higher-resolution phylogenetic analysis revealed that these isolates did not form distinct evolutionary branches but instead differentiated into distinct lineages. Some lineages shared the same ST as human isolates. However, these ST isolates cause rare infections in human clinical settings and do not belong to lineages prevalent in humans. Furthermore, analysis of the global animal *A. baumannii* population structure revealed the diversity of evolutionary lineages within animal *A. baumannii*. Among these diverse lineages, seven major IC lineages associated with human infections were identified, primarily isolated from companion animals such as horses, cats, and dogs. Other clonal lineages were mainly isolated from livestock, poultry, and wildlife. Notably, classification of isolates based on AMR gene load largely aligns with host source-based categorization. Higher-resolution core-genome-based phylogenetic tree revealed that *A. baumannii* isolates from companion animals share closer relationships with human isolates from the same regions than with those from livestock, poultry, or wildlife. Comparisons of resistance and virulence genes between animal and human isolates from the same region further support this finding.

## MATERIALS AND METHODS

### Isolation and identification of *A. baumannii*

In 2022, a total of 30 *A. baumannii* isolates were isolated from deceased embryos in Anhui province (duck, *n* = 26) and Hainan province (chicken, *n* = 4) in China (Table 1). These deceased poultry embryos originated from breeding companies in Anhui and Hainan provinces. They were culled due to failed hatching and sent to this laboratory for pathogen testing. To minimize the potential for environmental contamination during the isolation process, samples were specifically collected from intact brain tissue of unruptured embryos. The isolation of the bacteria was performed using MacConkey agar medium, while LB broth and agar were utilized for the subsequent propagation of all isolates. The identification of the species was conducted through matrix-assisted laser desorption/ionization time-of-flight mass spectrometry. Antimicrobial susceptibility testing was carried out using Mueller–Hinton agar medium.

**TABLE 1:** The information of 30 *A. baumannii* isolates from poultry^*a*^

Isolates	Accession number	Location	Host	Date (year.mo)	Company	CheckM2		ST	KL	OCL
						Completeness	Contamination			
ZWAb042	JBMBUQ000000000	Anhui	Duck	2022.06	Company A	100	0.17	77	KL175	OCL11
ZWAb043	JBMBUR000000000	Anhui	Duck	2022.06	Company A	100	0.04	2519	KL219	OCL3
ZWAb044	JBMBUS000000000	Anhui	Duck	2022.06	Company A	100	0.16	1858	KL219	OCL4
ZWAb045	JBMBUT000000000	Anhui	Duck	2022.06	Company A	100	0.66	**3328**	KL146	OCL12
ZWAb046	JBMBUU000000000	Anhui	Duck	2022.06	Company A	100	0.06	150	KL7	OCL6
ZWAb047	JBMBUV000000000	Anhui	Duck	2022.06	Company A	100	0.08	**3329**	KL203	OCL4
ZWAb048	JBMBUW000000000	Anhui	Duck	2022.06	Company A	100	0.06	374	KL125	OCL12
ZWAb049	JBMBUX000000000	Anhui	Duck	2022.06	Company A	100	0.03	**3330**	KL146	OCL12
ZWAb050	JBMBUY000000000	Hainan	Chicken	2022.11	Company B	100	1.27	**3331**	KL47	OCL6
ZWAb051	JBMBUZ000000000	Hainan	Chicken	2022.11	Company B	100	0.57	2115	KL42	OCL6
ZWAb052	JBMBVA000000000	Hainan	Chicken	2022.11	Company B	100	0.04	345	KL63	OCL6
ZWAb053	JBMBVB000000000	Hainan	Chicken	2022.11	Company B	100	0.44	1,102	KL190	OCL14
ZWAb054	JBMBVC000000000	Anhui	Duck	2022.10	Company C	100	0.03	150	KL7	OCL6
ZWAb055	JBMBVD000000000	Anhui	Duck	2022.10	Company C	100	0.34	**3332**	KL208	OCL16
ZWAb056	JBMBVE000000000	Anhui	Duck	2022.10	Company C	100	0.1	534	KL131	OCL1
ZWAb057	JBMBVF000000000	Anhui	Duck	2022.10	Company C	100	0.02	**3333**	KL155	OCL1
ZWAb058	JBMBVG000000000	Anhui	Duck	2022.10	Company C	100	0.06	108	KL14	OCL1
ZWAb059	JBMBVH000000000	Anhui	Duck	2022.10	Company C	100	0.02	**3333**	KL155	OCL1
ZWAb060	JBMBVI000000000	Anhui	Duck	2022.10	Company C	100	0.17	77	KL175	OCL11
ZWAb061	JBMBVJ000000000	Anhui	Duck	2022.10	Company C	100	0.06	374	KL125	OCL12
ZWAb062	JBMBVK000000000	Anhui	Duck	2022.10	Company C	100	0.06	374	KL125	OCL12
ZWAb063	JBMBVL000000000	Anhui	Duck	2022.10	Company C	100	0.17	77	KL175	OCL11
ZWAb064	JBMBVM000000000	Anhui	Duck	2022.10	Company C	100	0.13	**3335**	KL7	OCL7
ZWAb065	JBMBVN000000000	Anhui	Duck	2022.10	Company C	100	0.02	292	KL123	OCL1
ZWAb066	JBMBVO000000000	Anhui	Duck	2022.10	Company C	100	0.02	**3333**	KL155	OCL1
ZWAb067	JBMBVP000000000	Anhui	Duck	2022.10	Company C	100	0.06	108	KL14	OCL1
ZWAb068	JBMBVQ000000000	Anhui	Duck	2022.10	Company C	100	0.06	**3337**	KL221	OCL1
ZWAb069	JBMBVR000000000	Anhui	Duck	2022.10	Company C	100	0.06	**3337**	KL221	OCL1
ZWAb070	JBMBVS000000000	Anhui	Duck	2022.10	Company C	100	0.02	**3333**	KL155	OCL1
ZWAb071	JBMBVT000000000	Anhui	Duck	2022.10	Company C	100	0.02	**3333**	KL155	OCL1

^*a*^ The bold font indicates the ST types newly identified in this study, which have been submitted to the PubMLST database.

### Antibiotic susceptibility test

The Kirby–Bauer disk diffusion method was employed to ascertain the antimicrobial susceptibility of *A. baumannii* isolates. The antibiotic panel comprised the following agents: penicillins (piperacillin); β-lactam combination agents (ampicillin-sulbactam, piperacillin-tazobactam, and ticarcillin-clavulanate); cephems (ceftazidime, cefepime, and CTX); carbapenems (imipenem and meropenem); aminoglycosides (GM, tobramycin, and amikacin); tetracyclines (doxycycline, minocycline, and TE); fluoroquinolones (ciprofloxacin, kevofloxacin, and gatifloxacin); and folate pathway antagonists (trimethoprim-sulfamethoxazole). The quality control procedure employed utilized *Escherichia coli* ATCC 25922 for all antibiotics, with the resultant inhibition zone diameters being interpreted in accordance with the CLSI guidelines (M100 ED33-2023).

### Whole-genome sequencing and genome analysis

The genomic DNA extraction process was carried out utilizing the FastPure Bacteria DNA Isolation Mini Kit (DC103-01, Vazyme Biotech Co., Ltd). Whole-genome sequencing was conducted on the Illumina NovaSeq PE150 platform (Illumina, San Diego, CA, USA) at Beijing Novogene Bioinformatics Technology Co., Ltd. The sequencing reads were then assembled into contigs using Unicycler v5.0 (60). These assembled genomes undergo CheckM2 verification to assess their completeness and contamination levels (61). The capsule polysaccharides and lipo-oligosaccharide outer core have been identified as contributors to antibiotic resistance, virulence, and phage susceptibility of *A. baumannii*. The analysis of KL and OCL types was performed using Kaptive v3 (62). The investigation of plasmids and prophages, which have been identified as pivotal agents in bacterial environmental adaptation and evolution, was undertaken with a view to ascertaining their function in the horizontal transfer of virulence and antibiotic resistance genes. During the course of the assembly process, circularized DNA sequences were identified as complete plasmids and subsequently verified through the use of BLASTn analysis against the NCBI core_nt database. Phage identification across all isolates was performed using Phigaro v2.4.0 software (63). Plasmid identification and typing of 30 poultry embryo-sourced *A. baumannii* isolates were performed using SRST2 v0.2.0 (https://github.com/katholt/srst2) in conjunction with the acinetobacterplasmidtype_feb2025_V3 database of AcinetobacterPlasmidTyping (https://github.com/MehradHamidian/AcinetobacterPlasmidTyping). The corresponding replication initiation protein (Rep) gene sequences were extracted. AcinetobacterPlasmidTyping comprises a database of *Acinetobacter* plasmid Rep gene sequences corresponding to an updated plasmid rep typing scheme for *A. baumannii*. Redundant Rep gene sequences were removed using the CD-HIT Suite (https://github.com/weizhongli/cdhit), followed by multiple sequence alignment with MUSCLE (https://www.ebi.ac.uk/jdispatcher/msa/muscle). Based on the aligned sequences, a maximum likelihood phylogenetic tree was inferred using IQ-TREE v3.1.1 (https://iqtree.github.io/), with branch support assessed by 1,000 replicates of the Shimodaira–Hasegawa approximate likelihood ratio test and 1,000 ultrafast bootstrap replicates. The final tree was visualized using Chiplot (https://www.chiplot.online).

### Analysis of resistance and virulence genes

The genome scanning tool AbRicate v1.0.0 (https://github.com/tseemann/abricate) was utilized in the identification of antibiotic resistance and virulence genes. The genomes of all *A. baumannii* were subjected to a comprehensive search for virulence genes, employing the DNA sequences of the core data set in the VFDB database (64). Moreover, a comparable methodology was employed in the investigation of antibiotic resistance genes, utilizing the CARD database (65) and assigning predicted AMR phenotypes corresponding to the presence of AMR genes. A nucleotide BLAST search (https://blast.ncbi.nlm.nih.gov/Blast.cgi) was performed to determine whether ISAba1 (https://isfinder.biotoul.fr/scripts/ficheIS.php?name=ISAba1) is located immediately upstream of intrinsic genes such as *bla_ADC_* in all isolates, thereby rapidly assessing whether ISAba1-mediated upregulation underlies the observed changes in antibiotic susceptibility. Furthermore, a congruent methodology was utilized in the quest for virulence and AMR genes in prophages and plasmids.

### Molecular epidemiology

A total of 509 animal and 2,218 human *A. baumannii* genomes were retrieved from the NCBI GenBank database for the purpose of conducting a comprehensive analysis (Table S2). The present analysis was focused on two key objectives: firstly, to assess the distribution of *A. baumannii* in animals, and secondly, to undertake a comparative analysis of the genetic relationships between *A. baumannii* isolated from animal and human. The assignment of sequence types to all genomes was facilitated by the seven housekeeping genes of the Pasteur scheme of the PubMLST database (https://pubmlst.org), i.e., Pas_cpn60, Pas_fusA, Pas_gltA, Pas_pyrG, Pas_recA, Pas_rplB, and Pas_rpoB. Subsequently, MLST-based minimum spanning trees were constructed using GrapeTree’s modified algorithm (MSTree v2) to ascertain the distribution patterns of *A. baumannii* in different hosts (66). The genomes were annotated using the software Prokka v1.14.5 (67), and the GFF files that were generated from the annotation process were transferred to roary v3.13.0 (68) for pan-genomic analysis. Mafft v7 (69) was then used for core-genome comparison, and the output of this comparison was used for phylogenetic tree construction using FastTree v2.1 (70) based on maximum likelihood method. FastANI v1.34 is used to assess the similarity between pairs of genomes (https://github.com/ParBLiSS/FastANI). Finally, clinker (71) was used for comparative analysis of gene clusters, and Chiplot (https://www.chiplot.online) was used for plotting and landscaping of graphs.

### Statistical analysis

The statistical analysis was accomplished using the GraphPad Prism 9 software package. Test and control sets were compared by means of Student’s *t*-test. *P* values of <0.05 were considered statistically significant.

## Data Availability

Whole-genome sequencing data for this study are available from NCBI’s GenBank database (PRJNA1234050).

## SUPPLEMENTAL MATERIAL

Supplemental figures aem.00038-26-s0001.docx Fig. S1 to S10.

Table S1 aem.00038-26-s0002.xlsx Detailed information on the genomes of *A. baumannii* from different host sources, downloaded from NCBI.

Table S2 aem.00038-26-s0003.xlsx Diameter of tested antimicrobial agents to *A. baumannii* isolates.

ASM does not own the copyrights to Supplemental Material that may be linked to, or accessed through, an article. The authors have granted ASM a non-exclusive, world-wide license to publish the Supplemental Material files. Please contact the corresponding author directly for reuse.
