# Supplementary material for: The population structure of Acinetobacter baumannii isolated from animals: an emerging zoonotic threat
Source: Appl Environ Microbiol. 2026 Jun 15;92(7):e00038-26. doi: 10.1128/aem.00038-26 (PMC13399460; doi:10.1128/aem.00038-26)
Supplement: Supplemental figures — Fig. S1 to S10. [file aem.00038-26-s0001.docx]

SUPPLEMENTAL FILE 1


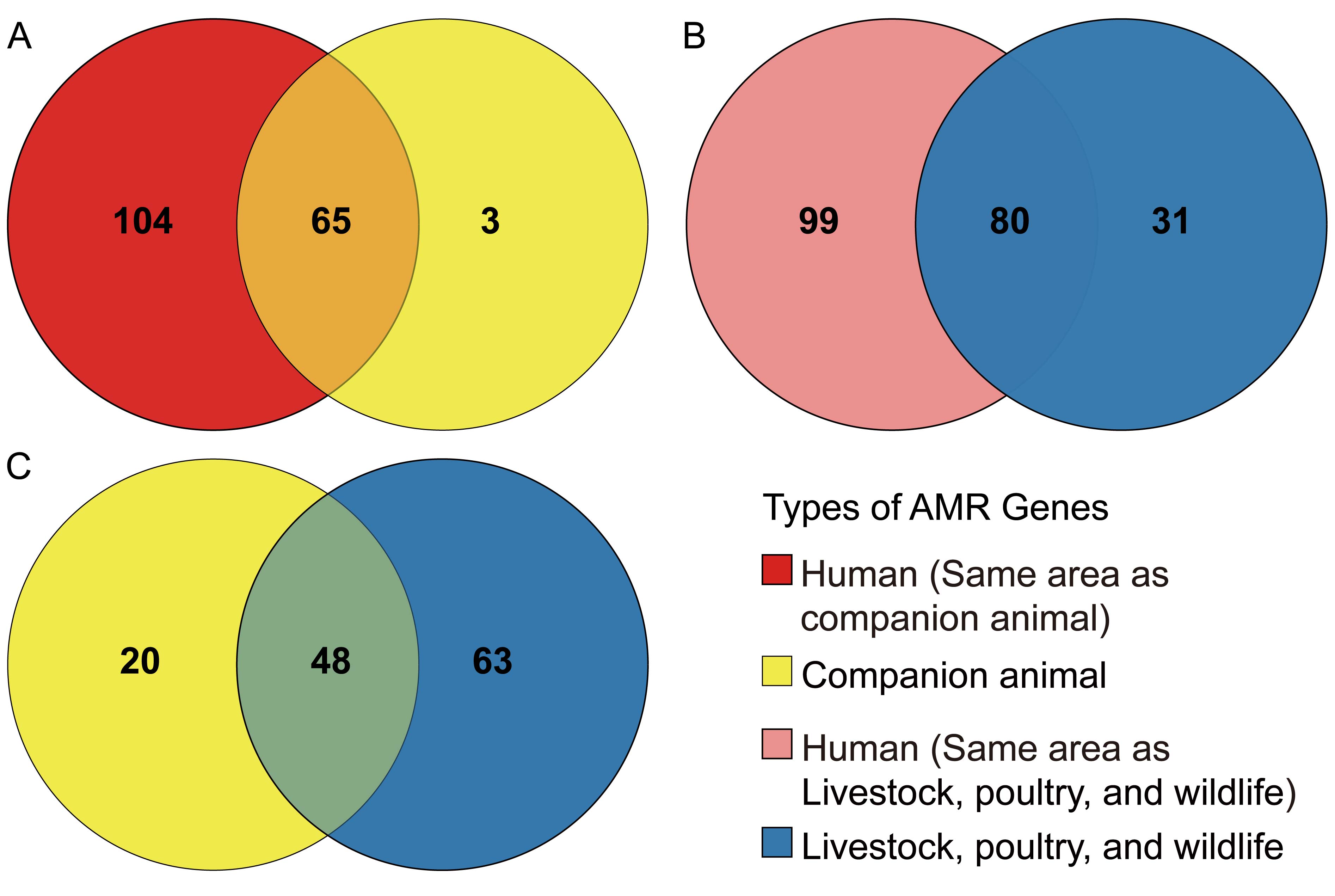


Fig S1 AMR genes shared between populations. (A) AMR genes carried by companion animal isolates and human isolates from the same region, including shared and unique types. (B) AMR genes carried by livestock, poultry, and wildlife isolates and human isolates from the same region, including shared and unique types. (C) AMR genes carried by companion animal isolates and livestock, poultry, and wildlife isolates, including shared and unique types.


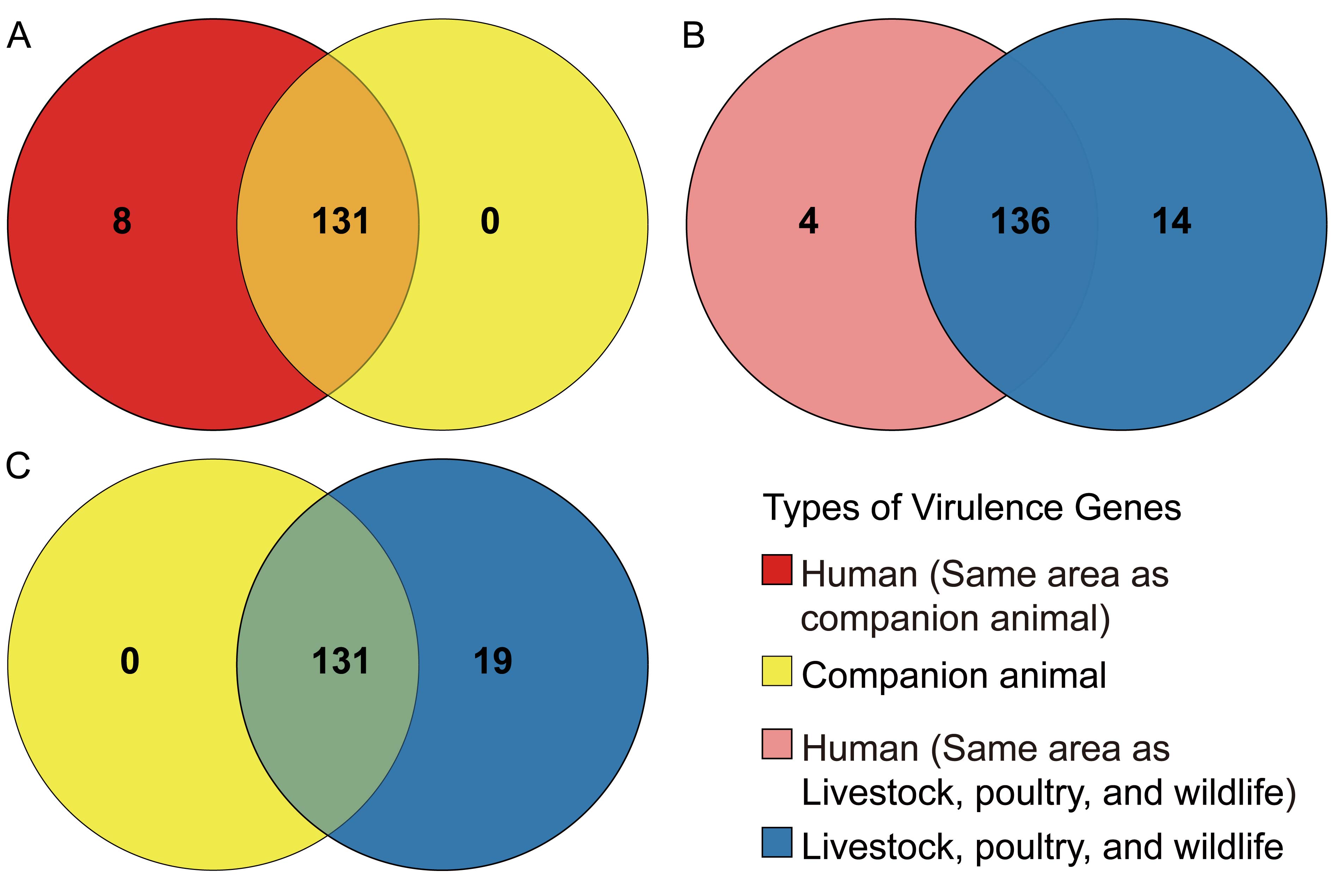


Fig S2 Virulence genes shared between populations. (A) Virulence genes carried by companion animal isolates and human isolates from the same region, including shared and unique types. (B) Virulence genes carried by livestock, poultry, and wildlife isolates and human isolates from the same region, including shared and unique types. (C) Virulence genes carried by companion animal isolates and livestock, poultry, and wildlife isolates, including shared and unique types.


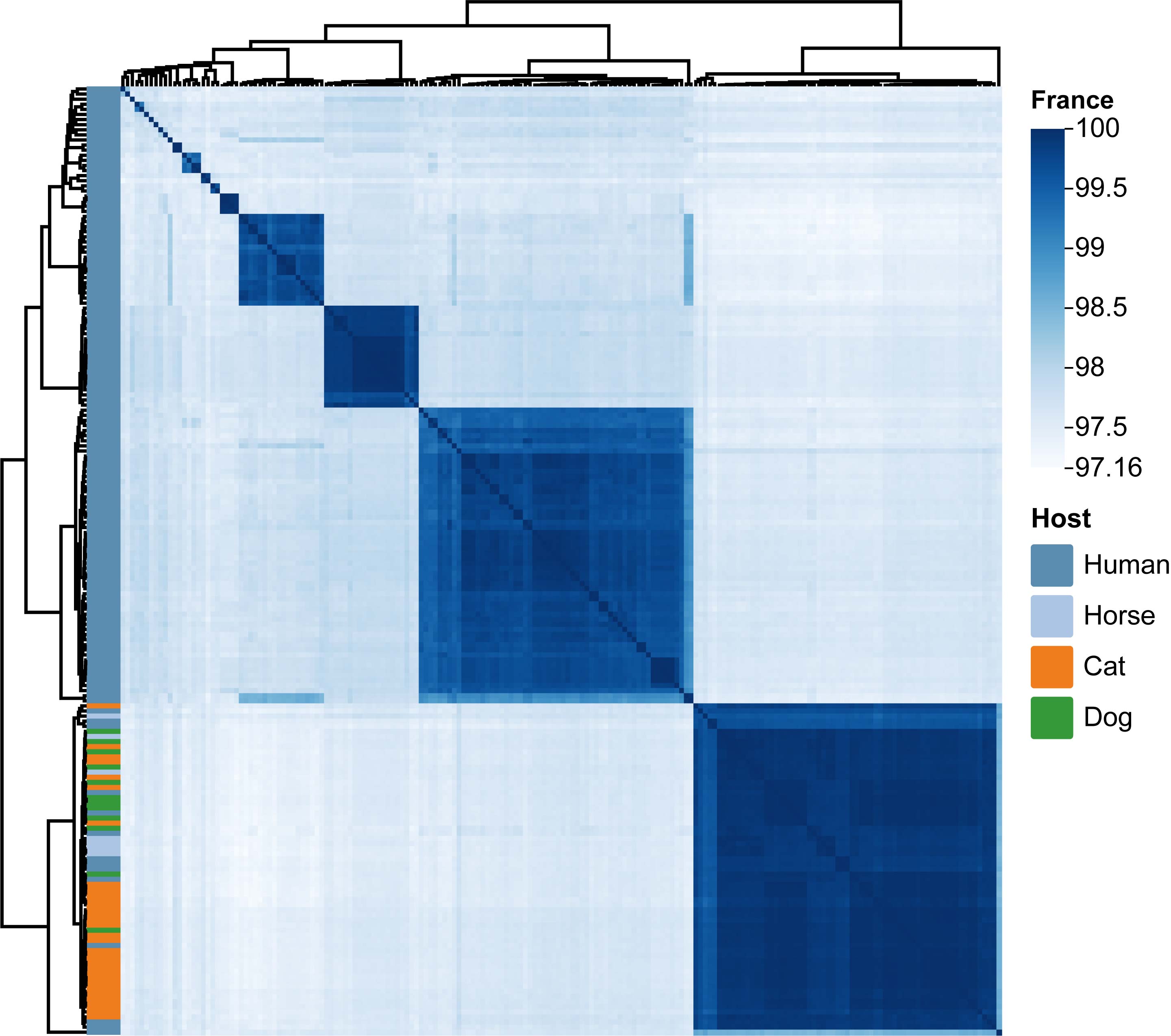


Fig S3 Genomic similarity between human isolates and animal isolates in France.


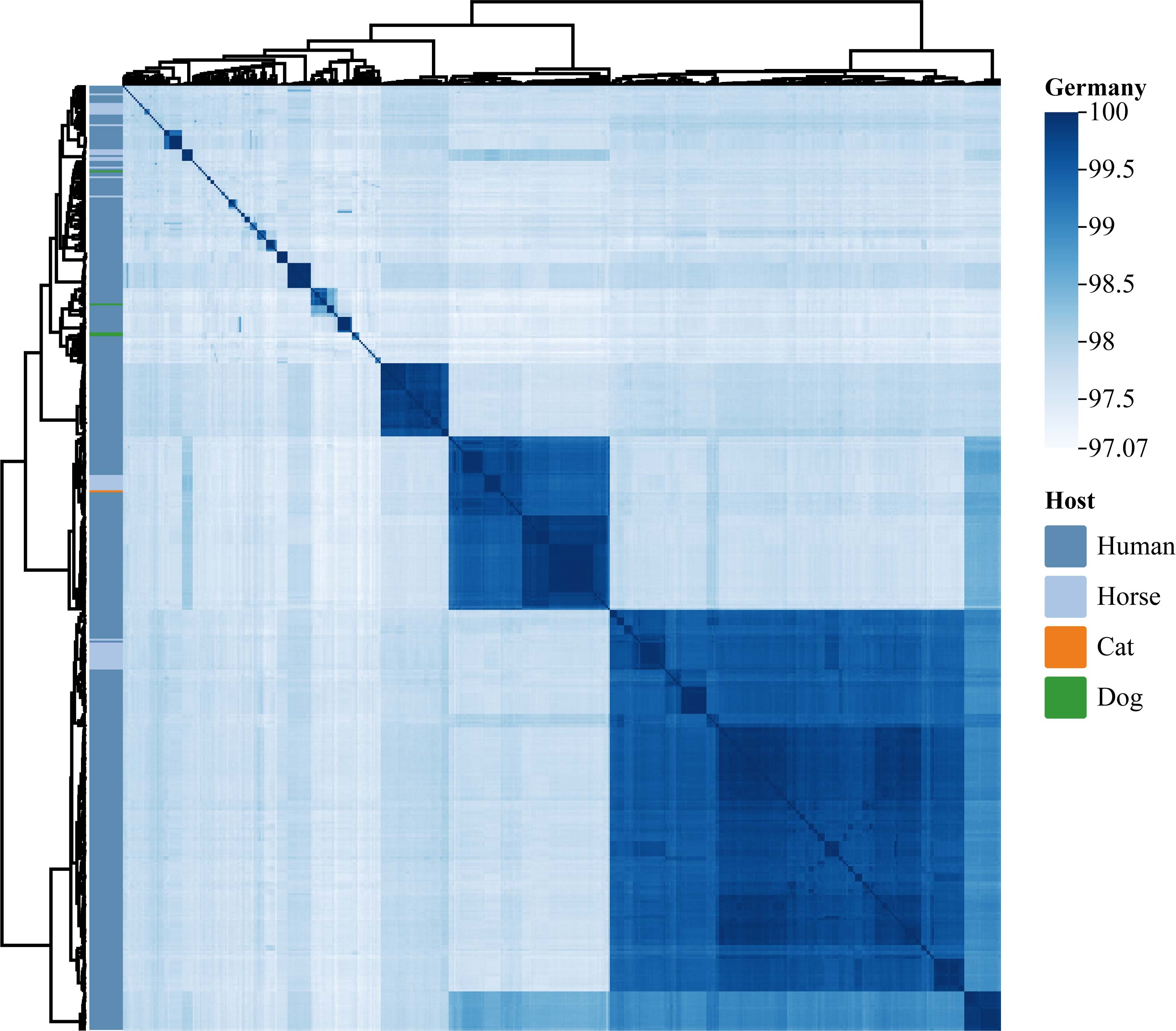


Fig S4 Genomic similarity between human isolates and animal isolates in Germany.


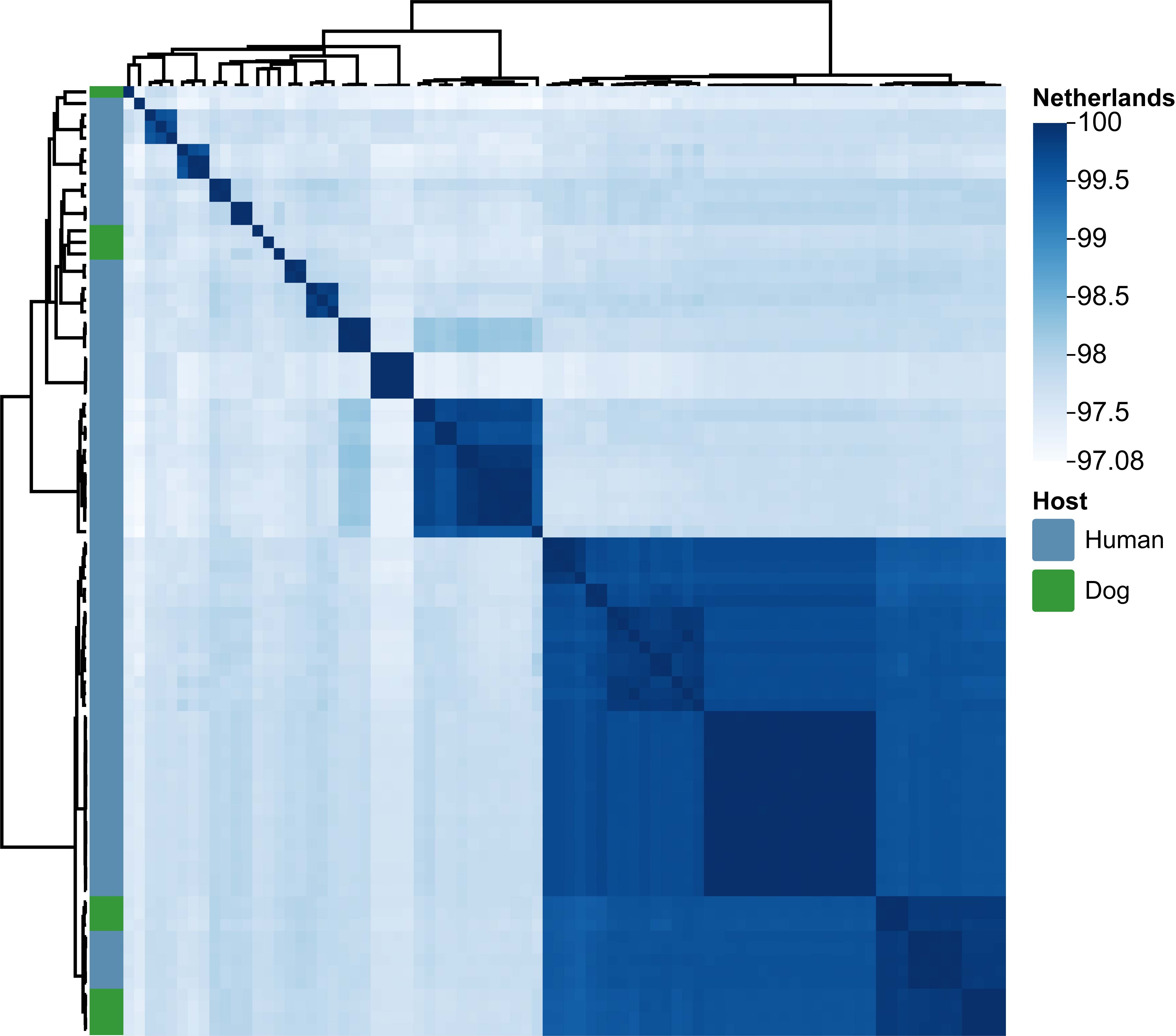


Fig S5 Genomic similarity between human isolates and animal isolates in the Netherlands.


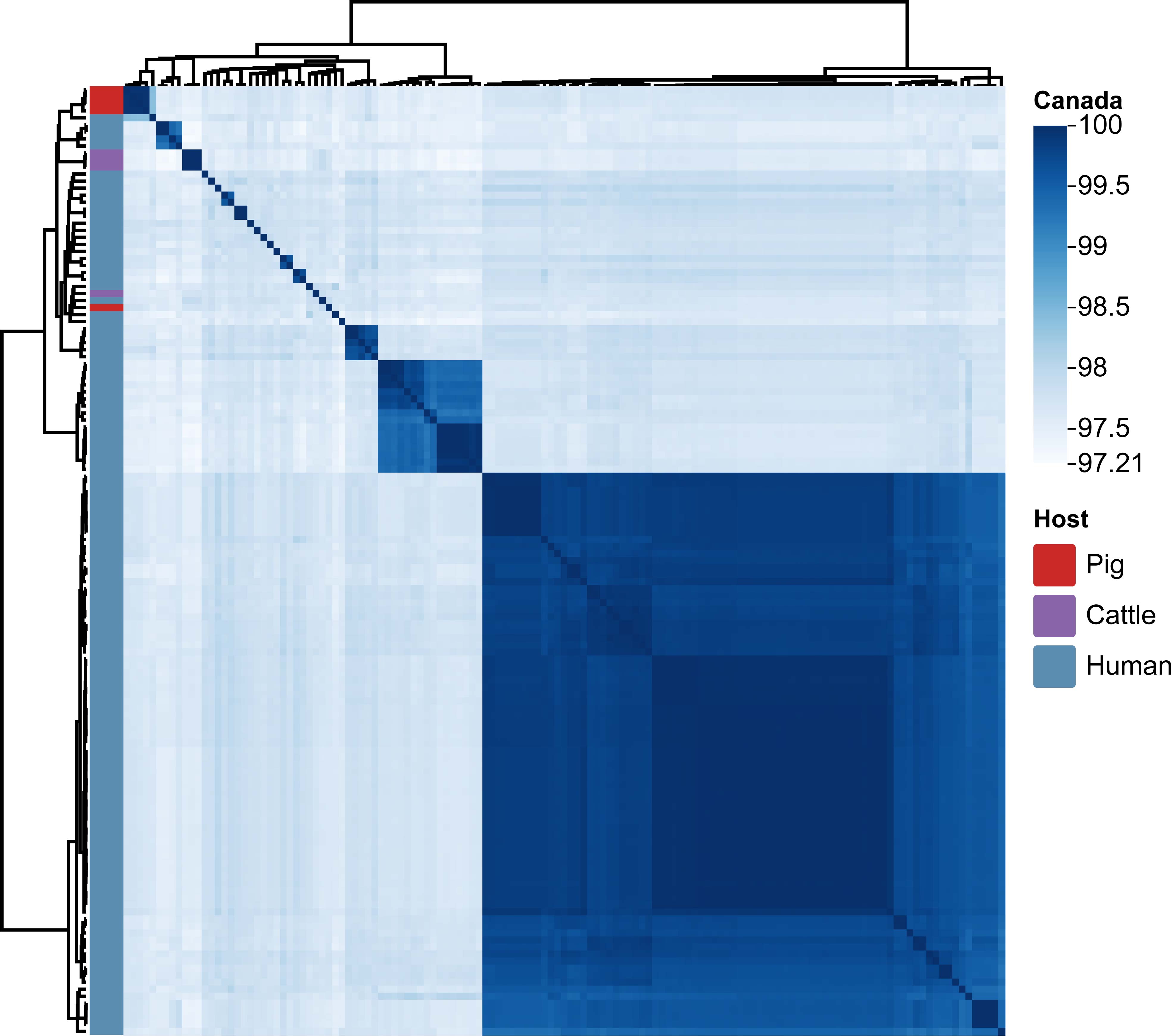


Fig S6 Genomic similarity between human isolates and animal isolates in Canada.


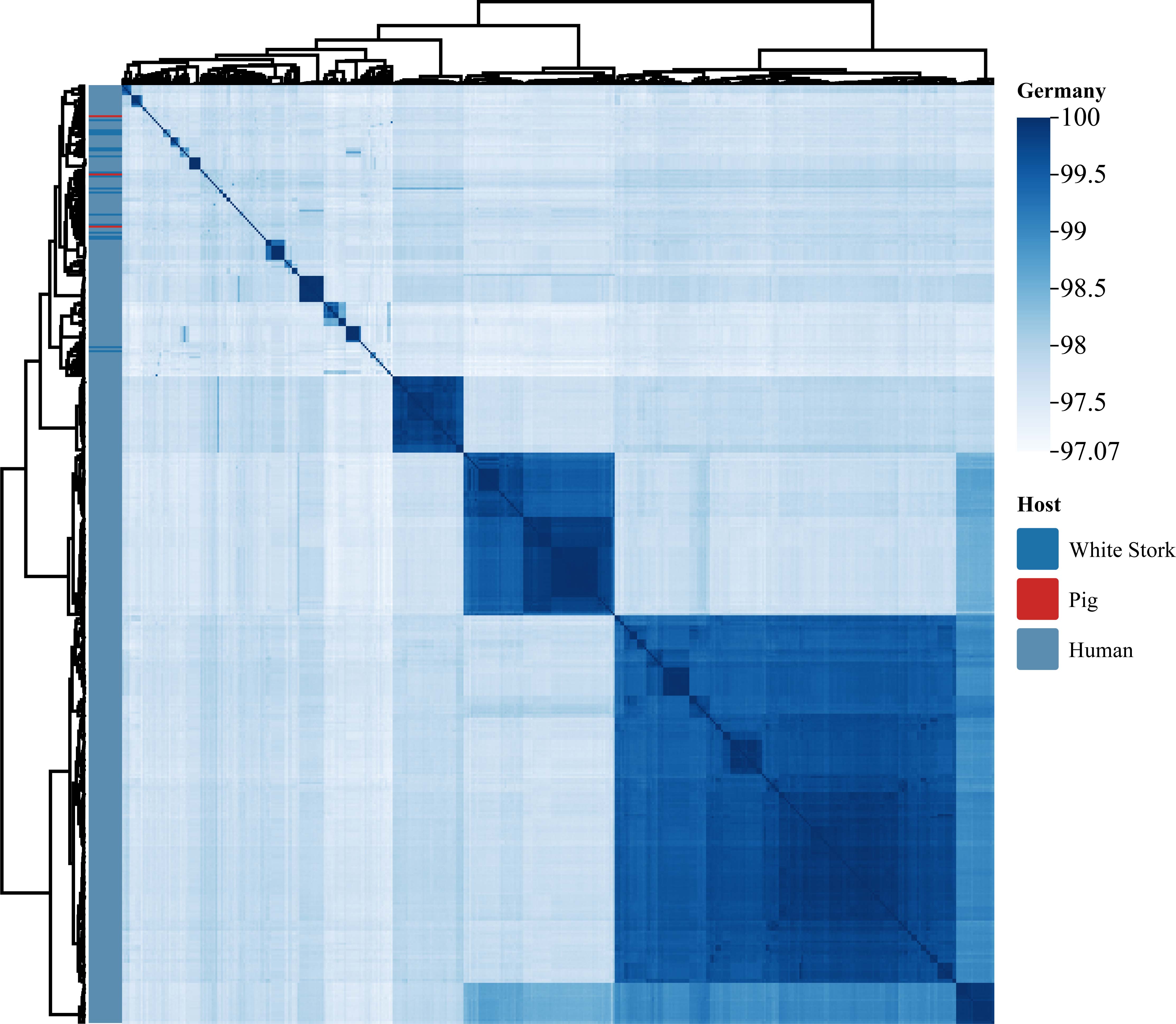


Fig S7 Genomic similarity between human isolates and animal isolates in Germany.


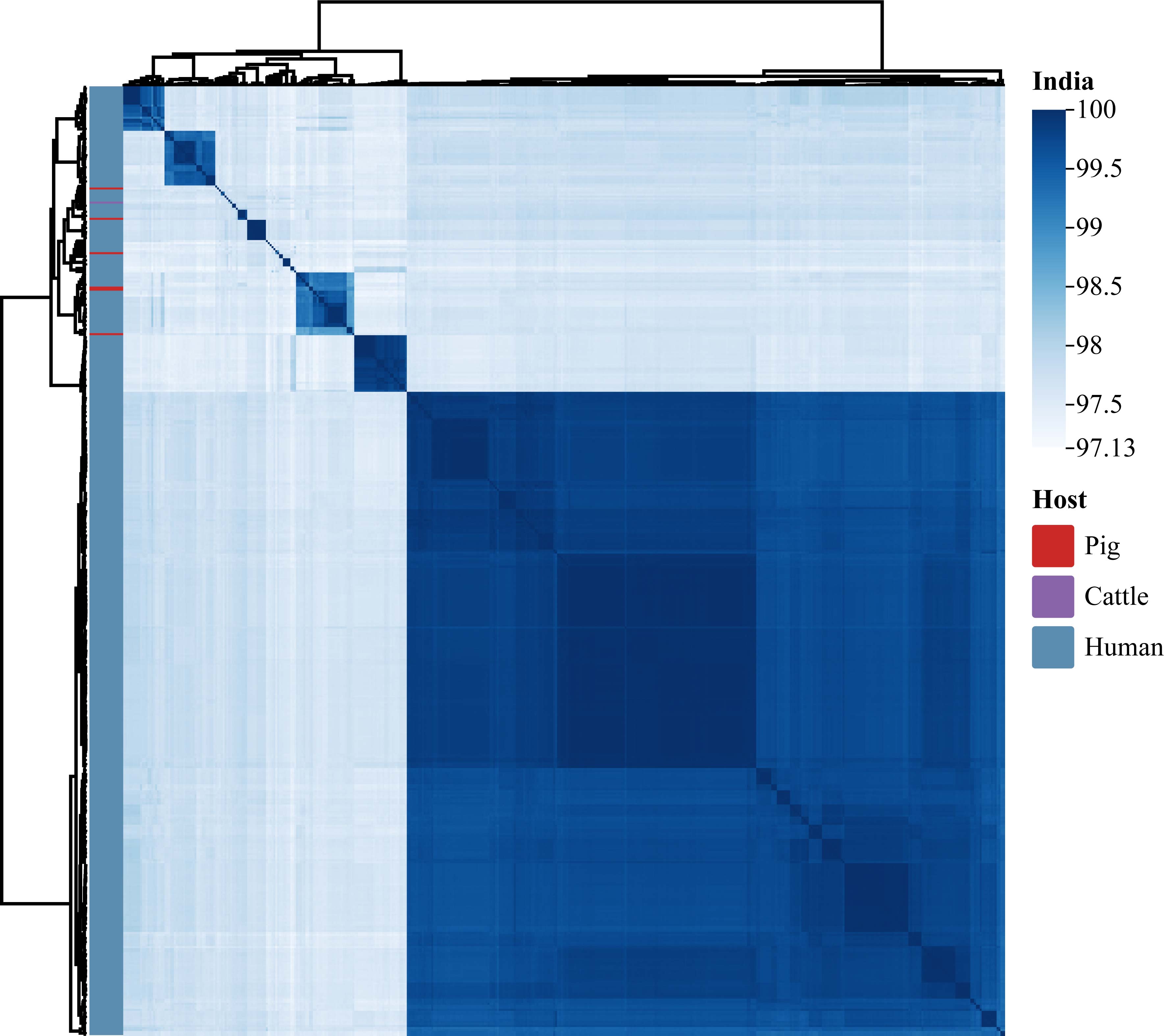


Fig S8 Genomic similarity between human isolates and animal isolates in India.


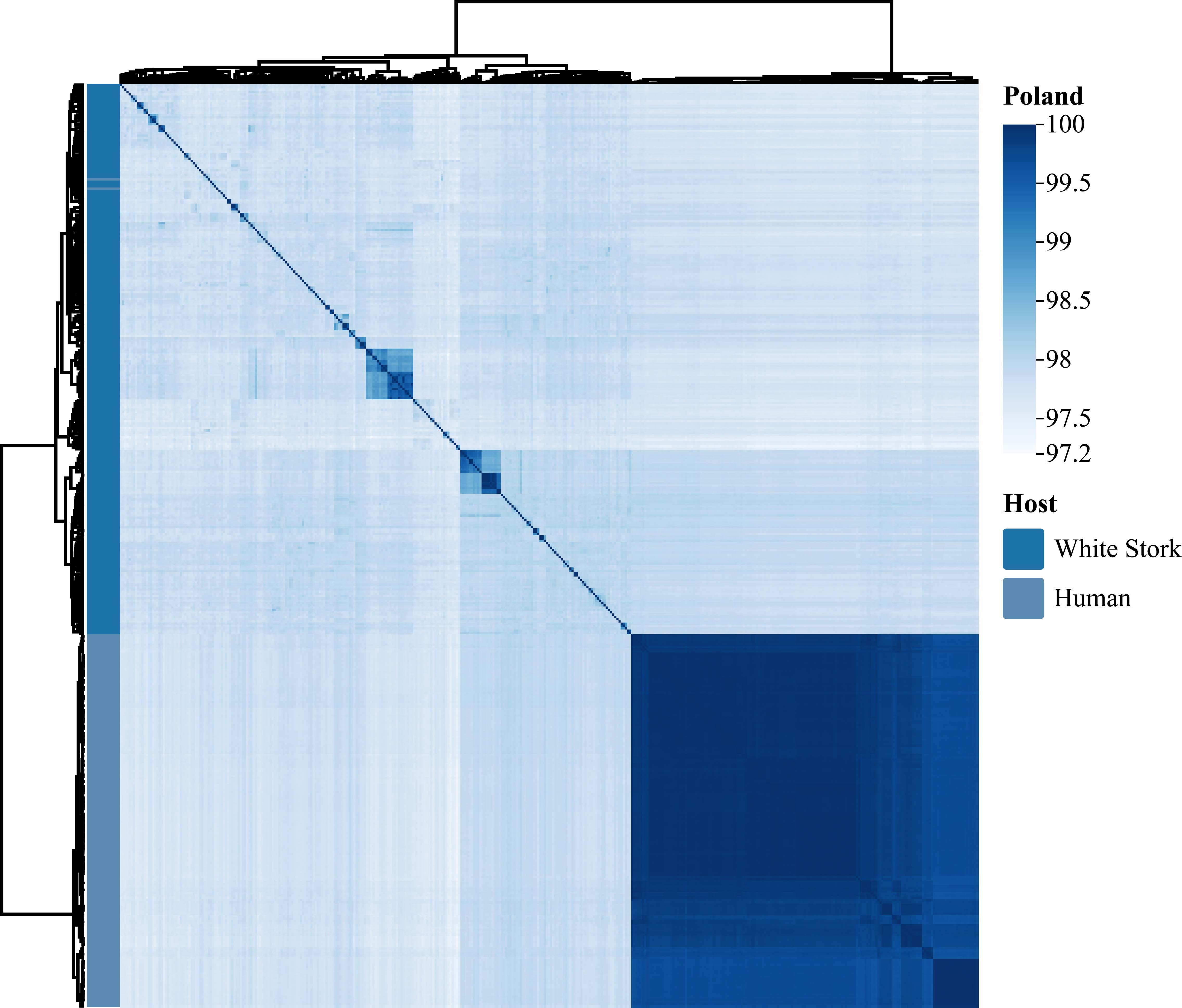


Fig S9 Genomic similarity between human isolates and animal isolates in Poland.


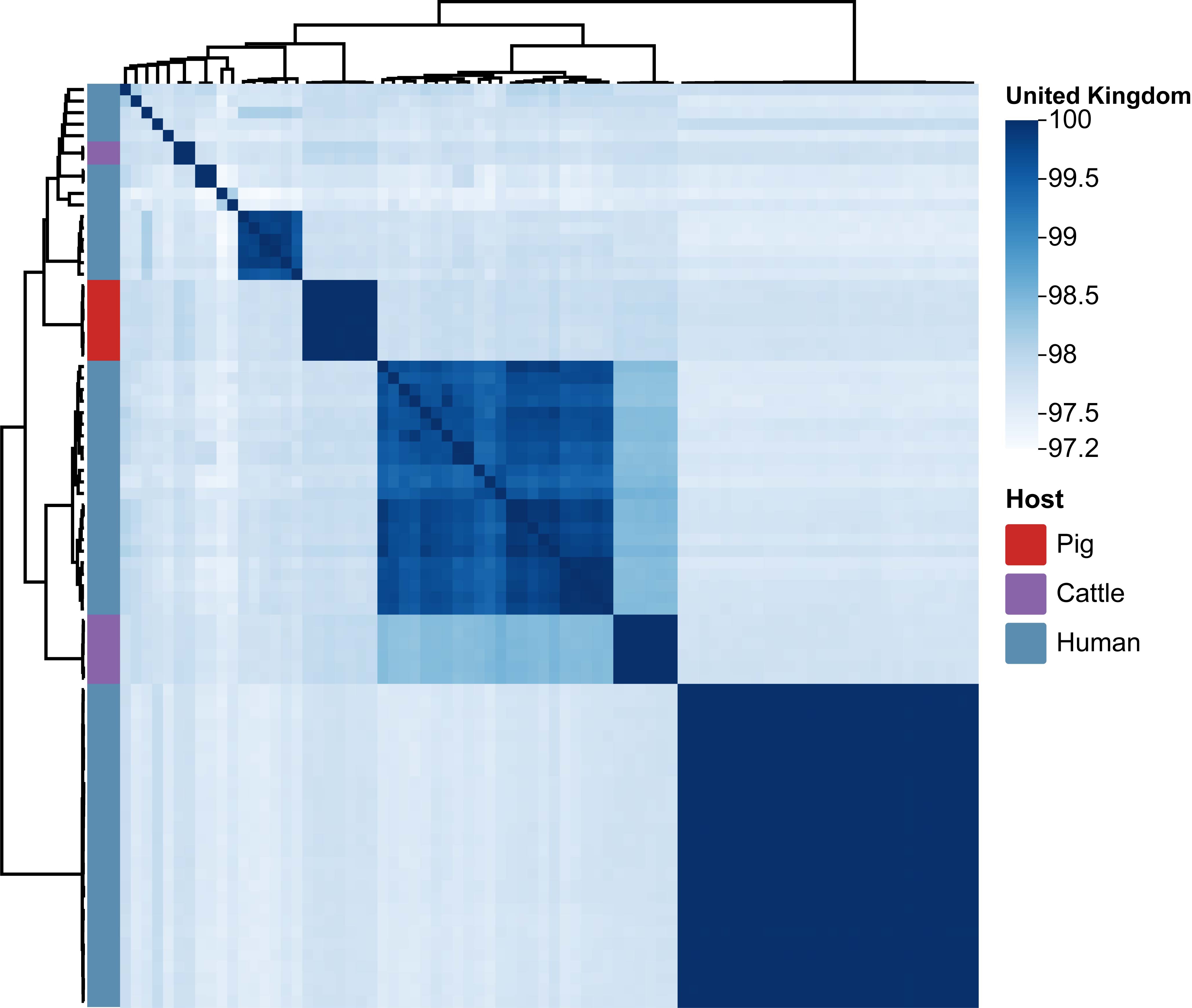


Fig S10 Genomic similarity between human isolates and animal isolates in the United Kingdom.
